# LTP Induction Boosts Glutamate Spillover by Driving Withdrawal of Perisynaptic Astroglia

**DOI:** 10.1016/j.neuron.2020.08.030

**Published:** 2020-12-09

**Authors:** Christian Henneberger, Lucie Bard, Aude Panatier, James P. Reynolds, Olga Kopach, Nikolay I. Medvedev, Daniel Minge, Michel K. Herde, Stefanie Anders, Igor Kraev, Janosch P. Heller, Sylvain Rama, Kaiyu Zheng, Thomas P. Jensen, Inmaculada Sanchez-Romero, Colin J. Jackson, Harald Janovjak, Ole Petter Ottersen, Erlend Arnulf Nagelhus, Stephane H.R. Oliet, Michael G. Stewart, U. Valentin Nägerl, Dmitri A. Rusakov

**Affiliations:** 1 UCL Queen Square Institute of Neurology, University College London, London WC1N 3BG, UK; 2 Institute of Cellular Neurosciences, Medical Faculty, University of Bonn, 53127 Bonn, Germany; 3 INSERM U1215, Neurocentre Magendie, 33000 Bordeaux, France; 4 Université de Bordeaux, 33000 Bordeaux, France; 5 Life Sciences, The Open University, Milton Keynes MK7 6AA, UK; 6 Institute of Science and Technology Austria, 3400 Klosterneuburg, Austria; 7 Institute of Basic Medical Sciences, University of Oslo, 0317 Oslo, Norway; 8 Karolinska Institutet, 171 77 Stockholm, Sweden; 9 Interdisciplinary Institute for Neuroscience, CNRS UMR 5297, 33000 Bordeaux, France; 10 German Center for Neurodegenerative Diseases (DZNE), 53175 Bonn, Germany; 11 Research School of Chemistry, Australian National University, Acton, ACT 2601, Australia; 12 EMBL Australia, Australian Regenerative Medicine Institute, Faculty of Medicine, Nursing and Health Science, Monash University, Melbourne, VIC 3800, Australia

**Keywords:** Excitatory synapse, long-term potentiation, glutamate spillover, perisynaptic astroglial processes, astrocyte plasticity, glutamate sensor imaging, super-resolution microscopy, hippocampus, whisker stimulation, barrel cortex

## Abstract

Extrasynaptic actions of glutamate are limited by high-affinity transporters expressed by perisynaptic astroglial processes (PAPs): this helps maintain point-to-point transmission in excitatory circuits. Memory formation in the brain is associated with synaptic remodeling, but how this affects PAPs and therefore extrasynaptic glutamate actions is poorly understood. Here, we used advanced imaging methods, *in situ* and *in vivo*, to find that a classical synaptic memory mechanism, long-term potentiation (LTP), triggers withdrawal of PAPs from potentiated synapses. Optical glutamate sensors combined with patch-clamp and 3D molecular localization reveal that LTP induction thus prompts spatial retreat of astroglial glutamate transporters, boosting glutamate spillover and NMDA-receptor-mediated inter-synaptic cross-talk. The LTP-triggered PAP withdrawal involves NKCC1 transporters and the actin-controlling protein cofilin but does not depend on major Ca^2+^-dependent cascades in astrocytes. We have therefore uncovered a mechanism by which a memory trace at one synapse could alter signal handling by multiple neighboring connections.

## Highlights

• Induction of synaptic LTP prompts withdrawal of perisynaptic astroglia
• The underlying mechanisms involve NKCC1 transporter and cofilin
• Reduced synaptic astroglial coverage boosts extrasynaptic glutamate escape
• LTP induction thus enhances NMDAR-dependent inter-synaptic cross-talk

## Teaser

Central synapses are often surrounded by thin astroglial processes that confine chemical neurotransmission to the synaptic cleft. Henneberger et al. find that memory trace formation at synaptic connections prompts withdrawal of these processes, thus boosting extrasynaptic neurotransmitter actions. Such actions can alter signal integration rules among neighboring synapses.

## Introduction

The surface of brain astroglia is packed with high-affinity GLT1 transporters that rapidly take up glutamate released by excitatory synapses (Danbolt, 2001; Verkhratsky and Nedergaard, 2018). GLT1-enriched perisynaptic astroglial processes (PAPs) that often surround synaptic connections (Grosche et al., 1999; Heller and Rusakov, 2015; Ventura and Harris, 1999) thus confine glutamate actions largely to the synaptic cleft. However, extrasynaptic glutamate escape, or “spillover,” can have a significant physiological impact. In the hippocampus, glutamate spillover has been causally related to a co-operative action of dendritic NMDA receptors (NMDARs) (Chalifoux and Carter, 2011; Hires et al., 2008), functional inter-synaptic cross-talk (Arnth-Jensen et al., 2002; Asztely et al., 1997; Lozovaya et al., 1999; Scimemi et al., 2004), heterosynaptic potentiation and depression (Vogt and Nicoll, 1999), and remote activation of metabotropic glutamate receptors (mGluRs) (Min et al., 1998; Scanziani et al., 1997). Glutamate escape underlies signaling between mitral cells in the olfactory bulb (Isaacson, 1999), and in the cerebellum between climbing fibers and interneurons (Coddington et al., 2013; Szapiro and Barbour, 2007) and between parallel fibers and stellate cells (Carter and Regehr, 2000). Changes in extrasynaptic glutamate signaling have also been related to cognitive decline (Pereira et al., 2014), fear conditioning (Tanaka et al., 2013; Tsvetkov et al., 2004), and heroin and cocaine relapse (Shen et al., 2014; Smith et al., 2017). However, whether the PAP-controlled glutamate spillover can be adaptively regulated by neural activity has remained unknown.

Astrocytes can generate molecular signals that regulate excitatory transmission (Araque et al., 2014; Bazargani and Attwell, 2016) and synaptic modifications associated with a memory trace (Adamsky et al., 2018; Henneberger et al., 2010; Min and Nevian, 2012; Shigetomi et al., 2013). Whether PAPs can also undergo activity-dependent remodeling has therefore been a long-standing question. Electron microscopy (EM) studies have reported increased astroglial coverage of synaptic samples that underwent induction of long-term potentiation (LTP) (Bernardinelli et al., 2014; Lushnikova et al., 2009; Wenzel et al., 1991) or in animals reared in complex environment (Jones and Greenough, 1996). In contrast, synaptic coverage by PAPs decreased following memory consolidation (Ostroff et al., 2014) or during lactation (Oliet et al., 2001). Nevertheless, EM cannot follow morphogenic events in time and might be prone to distortions of PAP morphology during tissue fixation (Korogod et al., 2015).

These factors necessitate evidence in live cells, which has been a challenge because of the nanoscopic size of PAPs. Several studies have elegantly used confocal or two-photon excitation (2PE) fluorescence microscopy to monitor fine changes in PAPs (Bernardinelli et al., 2014; Haber et al., 2006; Hirrlinger et al., 2004; Perez-Alvarez et al., 2014). However, dynamic fluorescent shapes seen in a light microscope are subject to interpretation. First, PAPs and inter-PAP distances are beyond the light diffraction limit, potentially giving rise to spurious shapes (Rusakov, 2015). Second, cell-permeable fluorescent tracers appear to underreport astroglial structure (Reeves et al., 2011). Finally, subtle re-distribution of the fluorescent label could be mistaken for changes in PAP shape or motility.

To avoid such uncertainties, we set out to monitor PAPs with microscopy methods that are not limited by diffraction of light, under several LTP induction protocols, in hippocampal slices and in the barrel cortex *in vivo*. We employed optical glutamate sensors to relate LTP-associated changes in PAPs to extrasynaptic glutamate escape. We identified key players in cell signaling cascades that could underpin such changes. The results thus unveil how a plasticity-inducing pattern of neural activity could trigger local PAP remodeling thus altering local rules of synaptic signal integration.

## Results

### LTP Induction Reduces PAP Volume

**Figure 1:**
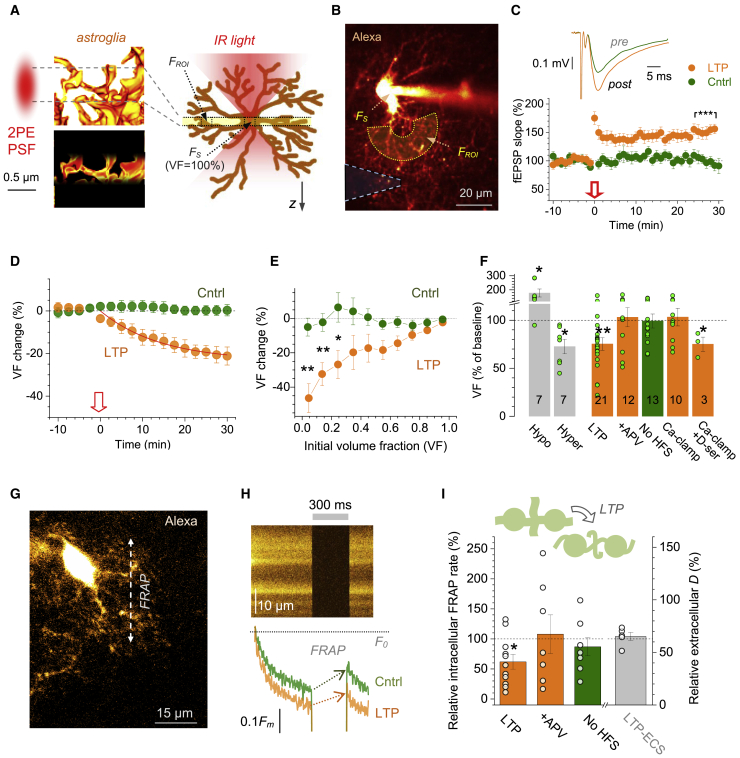
Reduced PAP Presence after LTP Induction at CA3-CA1 Synapses (A) Left: 2PE point-spread function (PSF) excites dye-filled PAPs (yellow, 3D EM fragment) within an ~1 μm focal plane (dotted lines; bottom). Right: fluorescence within ROI (*F*_*ROI*_) scales with PAP VF, reaching ~100% VF inside the 5–7 μm wide soma (*F*_*S*_). (B) Astrocyte filled with AF 594 (single focal section; λ_x_^2^^P^ = 800 nm); dashed cone, extracellular recording pipette. *F*_*ROI*_ and *F*_*S*_, areas of VF readout; see Video S1 for extended dynamic range. (C) Traces, *s. radiatum* fEPSPs, before (pre) and ~25 min after LTP induction (post); graph, relative fEPSP slope (mean ± SEM; arrow, induction onset); ^∗∗∗^p < 0.001 (25–30 min post-induction: 151.0% ± 6.7% compared to baseline, n = 18). (D) Relative change in PAP VF (%, mean ± 95% confidence interval [CI]) in control (green; n = 24 cells) and during LTP induction (arrow, onset; orange; n = 29); red line, best-fit exponential decay to steady state $VF\left(t\right)=VF_{ss}+\left(1-VF_{ss}\right)\exp\left(-t/\tau \right)$; *VF*_*ss*_ = 0.77 ± 0.04, τ = 14 ± 5 min. (E) Relative change in PAP VF (%, mean ± SEM) plotted against initial PAP VF, in control (n = 8 cells) and ~25 min after LTP induction (orange; n = 13; ^∗^p *<* 0.05, ^∗∗^p *<* 0.01, compared to control, df = 19). (F) Grey, PAP VF change (%, sample size n shown) in hypo-osmotic (220 mOsm/L) and hyper-osmotic (420 mOsm/L) solutions, as shown. Green and orange, PAP VF change 25–30 min after LTP induction in control (LTP, mean ± SEM: −25% ± 7%), in 50 μM APV (+APV, 3.1% ± 9.9%), with no HFS (−0.8% ± 7.3%), under Ca^2+^ clamp (Ca-clamp, 6.8% ± 9.5%), under Ca^2+^ clamp with 10 μM D-serine added (Ca-clamp^+^ D-ser, −24% ± 7%); ^∗∗^p < 0.01; ^∗^p < 0.05; dots, individual cells. (G) Evaluating diffusion coupling inside astroglia using FRAP of dialyzed AF 594 (STAR Methods; single focal section; ~80 μm depth); arrow, example line scan position. (H) Top: line scan as in (G) (baseline conditions; gray segment, shutter closed). Bottom: the corresponding fluorescence time course, before (Cntrl) and ~20 min after LTP induction (LTP); *F*_*0*_, initial intensity; arrows, FRAP during shutter-on period (full recovery takes 39–40 s). (I) Summary of FRAP tests (G and H); diagram, LTP induction may taper PAPs lowering diffusion coupling. Graph (mean ± SEM), FRAP rate relative to baseline (left ordinate): ~25 min after LTP induction (LTP, 62% ± 12%, n = 11; ^∗^p *<* 0.05); in 50 μM APV (108% ± 32%, n = 7); no-HFS control (87% ± 15%, n = 7). Grey (right ordinate), change in extracellular diffusivity ~25 min post-induction (LTP-ECS; 104% ± 7%, n = 5; Figures S1F–S1H); dots, individual tests.

First, we visualized astrocytes loaded whole-cell with Alexa Fluor 594 (AF 594), in CA1 *s. radiatum* of acute hippocampal slices, using 2PE. Here, fluorescence intensity *F*_*ROI*_ inside an ∼1 μm focal plane over an x-y region of interest (ROI), scales with the tissue volume fraction (VF) occupied by the dye-filled PAPs (Figure 1A, left). Because astrocyte territories do not overlap (Bushong et al., 2002), *F*_*ROI*_ represents all astroglia within the ROI. Thus, relating *F*_*ROI*_ to the fluorescence intensity *F*_*S*_ over the somatic region representing 100% VF (Figures 1A, right, S1A, and S1B) provides the local PAP VF readout, as detailed previously (Medvedev et al., 2014; Savtchenko et al., 2018). This readout gave average PAP VF of 6%–7% (cell bodies excluded), with or without gap junctions blocked (Figures S1B and S1C). A similar value was reported previously in area CA1 (Savtchenko et al., 2018) or *dentate gyrus* (Medvedev et al., 2014) and was in line with earlier EM data (Lehre and Rusakov, 2002; Patrushev et al., 2013).

Next, we induced LTP at CA3-CA1 synapses, with the classical protocol of high-frequency stimulation (HFS) applied to Schaffer collaterals, while monitoring PAP VF and local fEPSPs (Henneberger et al., 2010) (Figures 1B and 1C; STAR Methods). LTP induction prompted a gradual PAP VF decrease, with a time constant of ∼14 min and a projected steady-state value of ∼23% (Figure 1D; Video S1). No VF change occurred in control conditions (Figure 1D), ruling out confounding effects, such as dye photobleaching. Interestingly, ROIs with smaller initial PAP VF showed a stronger VF reduction (Figure 1E). Similar tests using EGFP-expressing astroglia showed a smaller effect, most likely due to restricted diffusion of EGFP compared to small AF 594 molecules, and no VF changes were detected after the induction of long-term depression (Figures S1D and S1E).

Video S1. Monitoring Volume Fraction of Astrocyte Processes during LTP Induction in Bulk, Related to Figure 1 Time-lapse series, example of an astrocyte in area CA1 loaded whole-cell with Alexa Fluor 594 (single optical section, λ_x_^2p^ = 810 nm, original recording at 16 bit gray-level). Two intensity ranges of the same time series are shown (left and right) to illustrate fluorescence time course at the soma (left) and within an example ROI (right, orange rectangle); orange rectangle, example ROI for PAP VF monitoring.

The VF decrease was blocked when LTP was suppressed, either by the NMDAR antagonist APV, or by clamping Ca^2+^ in the recorded astrocyte (Figure 1F) that inhibits astroglia-dependent release of the NMDAR co-agonist D-serine (Henneberger et al., 2010). In the latter test, LTP and VF reduction could be rescued by washing in 10 μM D-serine (Figure 1F), consistent with earlier reports (Adamsky et al., 2018; Henneberger et al., 2010). These results related VF reduction specifically to LTP induction rather than to HFS per se.

### LTP Induction Reduces Diffusion Coupling among Astroglial Processes

Fluorescence recovery after photobleaching (FRAP) of AF 594 can report diffusion coupling among astrocyte processes when the bleaching line-scan is applied in their midst (Figure 1G), as shown earlier (Anders et al., 2014; Savtchenko et al., 2018). In these tests, LTP induction slowed down FRAP kinetics, with no changes in control conditions (Figures 1H and 1I), suggesting reduced coupling among PAPs, possibly due to their partial shrinkage. At the same time, LTP induction had no effect on extracellular diffusion (Figure 1I) assessed with a fluorescence point-source method (Figures S1F–S1H) (Zheng et al., 2008). This was not surprising because CA1 astroglia occupy, soma excluded, only 6%–7% of the tissue volume (Figure S1C) (Savtchenko et al., 2018) of which 15%–20% is taken by the extracellular space (Syková and Nicholson, 2008). Thus, a 20%–30% decrease in PAP VF would increase local extracellular space by only 5%–10%.

### Stimulated Emission Depletion (STED) Imaging Reveals Decreased PAP Presence Near Spines upon LTP Induction

**Figure 2:**
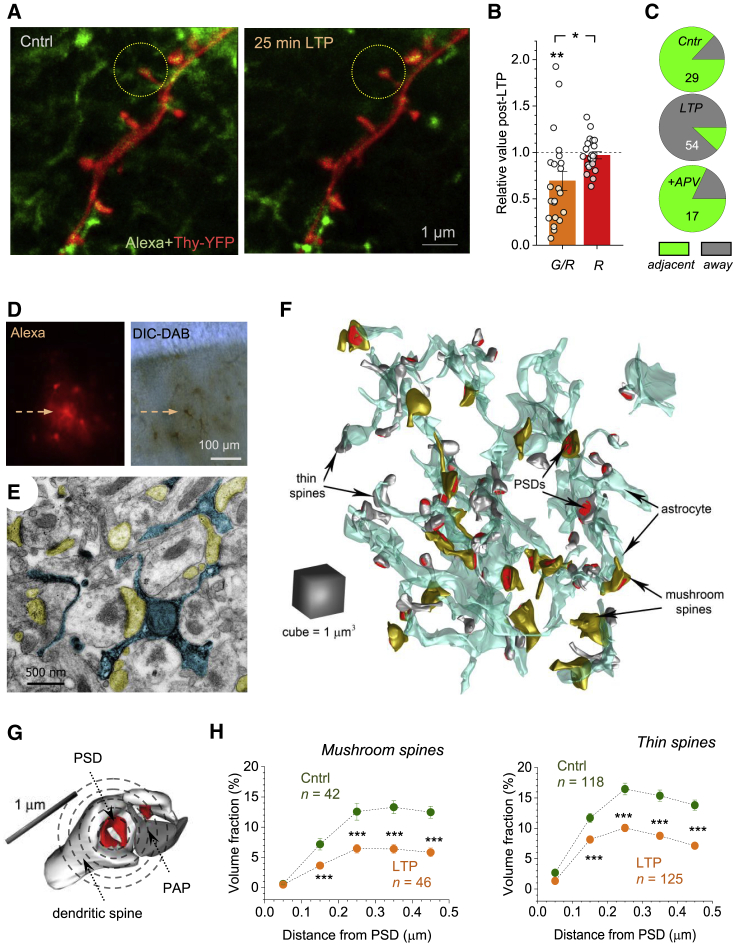
Live STED and Correlational 3D EM Report PAP Withdrawal after LTP Induction (A) STED images of dendritic spines (red, CA1 pyramidal cell; Thy1-YFP) and nearby astroglia (green; 600 μM AF 488), before and ~25 min after LTP induction, as indicated; circles, ROIs centered at spine heads. (B) LTP induction reduces the green/red (astroglia/neuron) pixel ratio within ROIs (G/R; mean ± SEM; 31% ± 10%, n = 22, ^∗∗^p *<* 0.01), with no effect on red pixel count (R; −3.1% ± 3.8%; ^∗^p < 0.02 compared to the G/R change, df = 42); dots, individual ROIs. (C) Proportion of dendritic spines that adjacent to (green) and away from (gray) PAPs, in control (Cntrl), 20–25 min post-induction (LTP), and the latter with 50 μM APV (+APV); spine numbers shown (Figures S2A–S2D). (D) Patched astrocyte loaded with biocytin (arrow, local astroglia stained through gap junctions), shown in the fluorescence (left) and DIC channel post-DAB conversion (right). (E) Electron micrograph showing PAPs of the patched astrocyte (arrow in D) filled with precipitate (blue), and adjacent dendritic spines (yellow) featuring PSDs. (F) Astrocyte fragment (cyan) reconstructed in 3D, including adjacent thin (white) and mushroom (yellow) dendritic spines with PSDs (red; Figure S2E). (G) Volumetric measure of synaptic astroglial coverage: PAP VF is calculated within 100 nm-thick concentric 3D shells (circles, not to scale) centered at the PSD (red). (H) PAP VF around PSDs (mean ± SEM) of thin and mushroom spines, in control and ~30 min after LTP induction, as indicated; sample sizes shown; ^∗∗∗^p < 0.001 (df = 86 for mushroom and df = 241 for thin spines).

STED microscopy enables monitoring live astroglia beyond the optical diffraction limit (Arizono et al., 2020; Panatier et al., 2014). We therefore turned to two-color STED (Tønnesen et al., 2018) combined with patch-clamp (Figures 2A and S2A) in organotypic slices. We used the Thy1-YFP transgenic mice and whole-cell AF 488 dialysis to image, respectively, CA1 pyramidal neurons and PAPs, in separate channels before and ∼20 min after LTP induction, with ∼70 nm x-y resolution (Figure 2A). Again, to avoid subjective judgement, we recorded the volumetric ratio of green-to-red pixels (G/R) within 1.5 μm ROIs centered at individual spine heads (Figure 2A). After LTP induction, G/R decreased by 31% ± 10% (n = 22, p < 0.001) (Figure 2B), thus corroborating results in acute slices (Figures 1A–1F). Stable red-pixel count (Figure 2B) ruled out Thy1-YFP photobleaching whereas AF 488 bleaching was prevented by dialysis.

STED images revealed subtle changes in some dendritic spines during LTP (Figure S2B). To explore this further while minimizing STED-induced photodamage, we compared randomized groups of spines. The LTP group had a much smaller fraction of the PAP-contacting spines (Figure 2C), larger heads in the no-PAP-contact spines, and a greater fraction of distinctly large spine heads (>500 nm wide, 12/54) overall, compared to control (3/29, Figures S2C and S2D).

### Correlational 3D EM Shows Reduced Occurrence of PAPs after LTP Induction

To understand further changes on the nanoscale, we turned to correlational 3D EM. We loaded an astrocyte with AF 594 and biocytin (Figure 2D), either in baseline conditions or 15–20 min after LTP induction, followed by rapid slice submersion into fixative and DAB conversion for EM (Figures 2D and 2E; STAR Methods). The embedded slices were cut into 60–70 nm serial sections, the patched astrocyte was located (Figures 2D and 2E), and its fragment with the adjacent synapses were 3D-reconstructed from 200–300 sections (Figures 2F and S2E), as detailed earlier (Medvedev et al., 2014; Savtchenko et al., 2018).

To evaluate synaptic PAP coverage volumetrically, we calculated PAP VF inside 100 nm thick concentric spherical shells centered at individual postsynaptic densities (PSDs) (Figure 2G; STAR Methods), up to ∼0.5 μm, the average nearest-neighbor distance between CA3-CA1 synapses. Although “thin” and “mushroom” spines have distinct identities (Matsuzaki et al., 2001), we found that LTP induction reduced local PAP VF for both types (Figure 2H). Here, VF values agreed with the earlier EM data (Lehre and Rusakov, 2002; Patrushev et al., 2013) and 2PE data (Medvedev et al., 2014; Savtchenko et al., 2018) (Figure S1C), arguing that our EM results are unlikely to be biased by fixation (Korogod et al., 2015) (see Discussion).

### LTP-Induced PAP Withdrawal Depends on Activation of NKCC1

**Figure 3:**
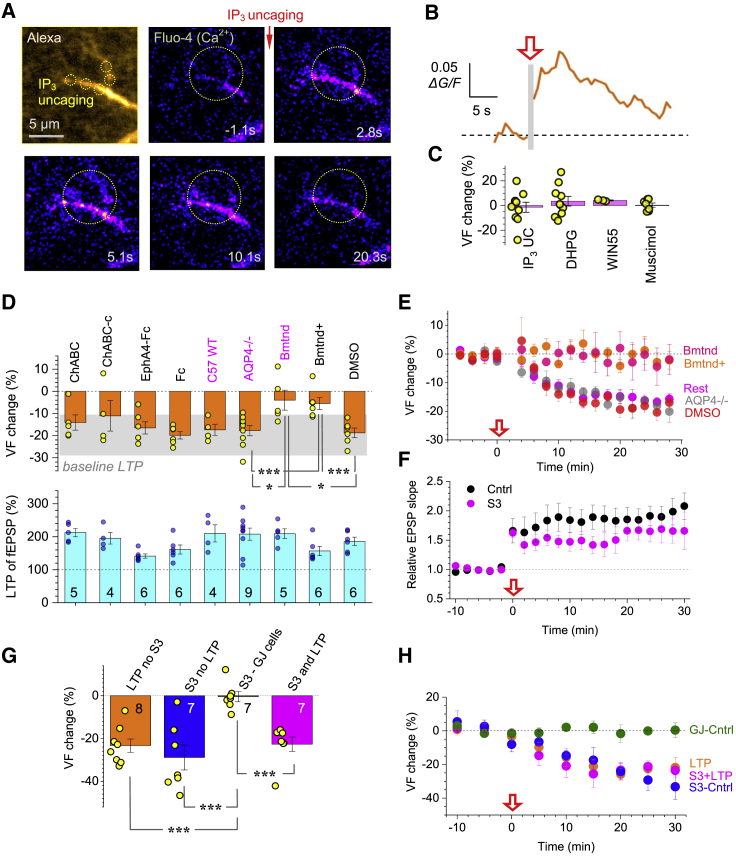
LTP-Associated PAP Withdrawal Depends on NKCC1 and Cofilin (A) Top left: astrocyte fragment (5 μm z stack); circles, uncaging spots (400 μM NPE-IP_3_; AF 594 channel, λ_x_^2P^ = 840 nm). Other panels: Ca^2+^ response (200 μM Fluo-4; false colors) to IP_3_ spot-uncaging (at t = 0; five 5 ms pulses at 5 Hz; λ_u_^2P^ = 720 nm); time lapse shown; circle, ROI for Ca^2+^. (B) Time course of intracellular Ca^2+^ signal (Δ*F*/*G*) in ROI shown in (A); one-cell example; red arrow (gray segment), IP_3_ uncaging. (C) Relative change in PAP VF (%, mean ± SEM) 25 min after: spot-uncaging of intracellular IP_3_ (−1.4% ± 4.1%, n = 10), application of DHPG (300 μM, 3.5% ± 3.9%, n = 10), CB1 receptor agonist WIN55 (1 μM, 4.1% ± 0.4%, n = 3), or GABA receptor agonist muscimol (20 μM, 0.4% ± 1.7%, n = 6). (D) Relative change in PAP VF (%, mean ± SEM; top) ~25 min after LTP induction, and the corresponding LTP level (%, mean ± SEM; bottom, sample size shown): in the presence of 0.5–0.7 U/mL chondroitinase ABC (ChABC, −14% ± 3%), control ChABC-c (−11% ± 7%), 10 μg/mL EphA4-Fc (−17% ± 3%), 10 μg/mL Fc control (−20% ± 2%), wild-type C57BI6 mice (−17% ± 3%), AQP4^−/−^ knockout mice (−18% ± 2%), 20 μM intracellular bumetanide (Bmtnd, −4% ± 4.5%), 50 μM intracellular bumetanide + 100 μM extracellular TGN-020 (Bmtnd^+^, −5.5% ± 2.7%), DMSO control 0.2% external + 0.05% internal (−19% ± 2%); blue text, data from mice; gray shadow, 95% CI for PAP VF change after LTP induction in control conditions; ^∗^p < 0.02 (df = 12 for AQP4^−^/ versus Bmtnd, df = 9 for Bmtnd versus DMSO), ^∗∗∗^p < 0.005 (df = 13 for AQP4^−^/ versus Bmtnd^+^, df = 10 for Bmtnd+ versus DMSO; t test or Mann-Whitney independent sample tests). (E) PAP VF change (%, mean ± SEM) during LTP induction (arrow) in key tests shown in (D), as indicated, and summary for other experiments (Rest). (F) Relative fEPSP slope (mean ± SEM) during LTP induction at CA3-CA1 synapses in control (n = 10) and with S3 peptide inside astroglia (200 μM , n = 6), as shown (Figures S3B and S3C). (G) Occlusion experiment: PAP VF change (%, mean ± SEM, sample size shown): ~25 min after LTP induction in control (LTP no S3; −23% ± 3%), no LTP induction, whole-cell loaded S3 (S3 no LTP; −29% ± 6%); same but recorded in gap-junction connected astrocytes devoid of S3 (S3-GJ cells; −0.4% ± 2.4%); and ~25 min after LTP induction with S3 (S3 and LTP; −27% ± 3%); ^∗∗∗^p < 0.001 (df = 13 for “LTP no S3” versus “S3 GJ Cells,” df = 12 for the rest). (H) Time course of PAP VF (%, mean ± SEM) in the occlusion experiments shown in (G); notations as in (G).

To explore cellular mechanisms underlying PAP withdrawal, we first examined major astroglial Ca^2+^-signaling cascades that engage mGluRs and IP_3_ receptors (Porter and McCarthy, 1997; Volterra et al., 2014) and can alter PAP motility (Perez-Alvarez et al., 2014). We spot-uncaged IP_3_ inside cell branches: this evoked local Ca^2+^ rises (Figures 3A and 3B) but had no effect on PAP VF (Figure 3C). Puff application of the group I mGluR agonist DHPG had a similar outcome (Figure 3C), and PAP VF remained unaffected by WIN55, an agonist of the cannabinoid CB1 receptor that is active in astroglia (Navarrete and Araque, 2010). Similarly, the GABA_A_ receptor agonist muscimol, which triggers slight shrinkage of sulforhodamine-101 stained astroglia (Florence et al., 2012), had no effect on PAP VF (Figure 3C).

We next tested morphogenic agents associated with synaptic remodeling. However, removing the extracellular matrix (ECM) chondroitin sulfate (Dityatev and Schachner, 2003) with chondroitinase ABC (Kochlamazashvili et al., 2010), or blocking the ephrin/EphA4 cascade (Filosa et al., 2009; Murai et al., 2003; Nishida and Okabe, 2007) with EphA4-Fc had no effect on the LTP-induced PAP VF reduction (Figure 3D).

We next turned to ion and water exchange mechanisms, in which aquaporin-4 (AQP4) plays a prominent role (Nagelhus and Ottersen, 2013). However, the LTP-associated reduction in PAP VF remained intact in the AQP4 knockout (KO) mice (Thrane et al., 2011). Another key player in cell volume regulation is the Na^+^-K^+^-2Cl^−^ cotransporter NKCC1, which is widely expressed in astroglia (Hoffmann et al., 2009; Kaila et al., 2014). To ensure single-cell specificity, we loaded astrocytes whole-cell with the NKCC1 blocker bumetanide (20 μM). Strikingly, bumetanide blocked the reduction of PAP VF while preserving LTP induction (Figures 3D and 3E, *Bmtnd*) whereas in baseline conditions bumetanide had no effects on PAP VF per se (Figure S3A). The NKCC1 involvement was confirmed in rats with 50 μM intracellular bumetanide (Figures 3D and 3E, *Bmtnd*^+^); here, the AQP4 blocker TGN-020 (Igarashi et al., 2011) was added to the bath, to “mimic” AQP4 KO (although see Tradtrantip et al., 2017).

### Activating Cofilin Cascade Occludes LTP-Induced Changes in PAPs

In glioblastoma cells, NKCC1 provides a protein scaffold regulating the phosphorylation of cofilin-1 (Schiapparelli et al., 2017), and in neurons, transporter KCC2 plays a similar role (Llano et al., 2015). Cofilin-1 is a pH-dependent regulator of actin filament polymerization, which in turn controls remodeling of thin cell protrusions (Bravo-Cordero et al., 2013; Ethell and Pasquale, 2005). To probe this cascade, we dialyzed astroglia with peptide S3, a specific inhibitor of cofilin-1 phosphorylation (Aizawa et al., 2001; Liu et al., 2016). Unlike bumetanide, this preserved both LTP induction and the PAP VF decrease (Figures 3F, S3B, and S3C; STAR Methods). However, peptide S3 dialysis reduced PAP VF by 20%–25% in baseline conditions, too, similar to the LTP case (Figure 3G): here, astrocytes connected to the patched cell via gap junctions (impermeable to S3, MW ∼1.5 kDa) showed no PAP changes, confirming a cell-specific action (Figure 3G). Furthermore, when we combined LTP induction with S3 dialysis (Figures S3B and S3C), the kinetics of PAP shrinkage were the same as under LTP induction alone or under S3 dialysis alone (Figure 3H). Thus, peptide S3 action occluded the effect of LTP induction on PAP VF, suggesting a shared mechanism (see Discussion).

### Single-Synapse LTP Induction Prompts Local PAP Retraction

**Figure 4:**
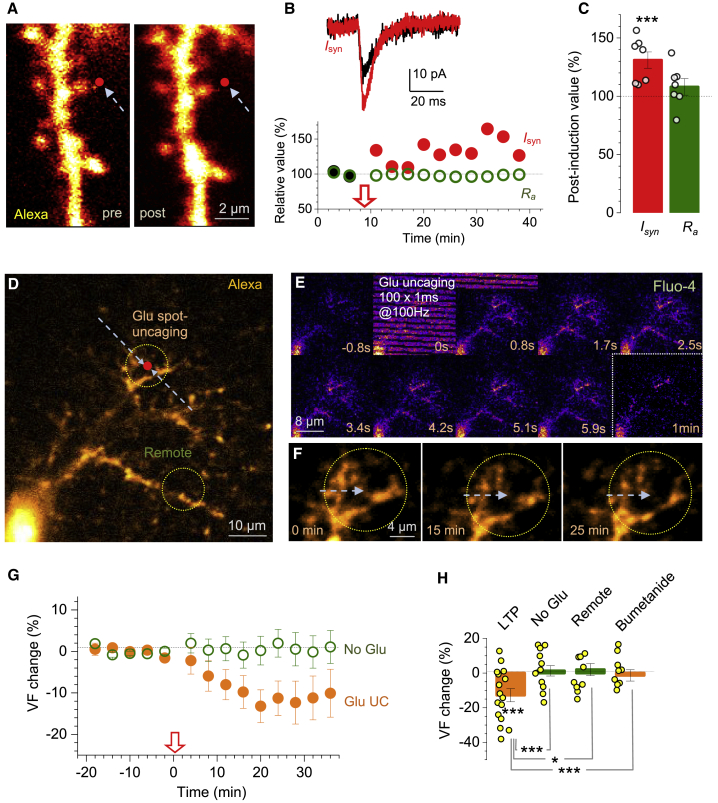
LTP Induction at Individual CA3-CA1 Synapses Reduces Local PAP Presence (A) Dendritic fragment, CA1 pyramidal cell (AF 594 channel), showing glutamate uncaging spot (red dot; 2.5 mM bath-applied MNI-glutamate) before (pre) and ~20 min after spot-uncaging LTP induction (post). (B) One-spine example. Traces, EPSCs (*I*_*syn*_, voltage-clamp) during baseline (black) and ~30 min after LTP induction (red; see Figures S4A and S4B for Ca^2+^ dynamics). Graph, relative EPSC amplitude (*I*_*syn*_; black and red circles) and cell access resistance (*R*_*a*_, green) time course; arrow, LTP induction onset. (C) Statistical summary of experiments in (A) and (B) (mean ± SEM; n = 7, ^∗∗∗^p < 0.005); notations as in (B); dots, individual tests. (D) Example, astrocyte fragment (whole-cell AF 594, single focal section); red dot, glutamate uncaging spot; circles, ROIs for PAP VF monitoring near the spot and away, as shown. (E) Time-lapse frames (area shown in D): astrocyte Ca^2+^ response (Fluo-4, λ_x_^2P^ = 840 nm) to the spot-uncaging LTP protocol (λ_u_^2P^ = 720 nm). (F) Astrocyte fragment near the uncaging spot (as in D; arrow) immediately after (0 min), at 15 min and 25 min after LTP induction (~9 μm z stack average); PAP retraction seen at 15–25 min (Figures S4C–S4E; Video S2). (G) PAP VF change (%, mean ± SEM) in tests shown in (D) and (E) (Glu, n = 11), and with no MNI-glutamate (no Glu, n = 11; arrow, uncaging onset). (H) Summary: PAP VF change (%, mean ± SEM) ~25 min post-induction (LTP, −13% ± 4%, ^∗∗∗^p < 0.005, n = 16), with no MNI-glutamate (no Glu, 1.3% ± 3.0%, n = 9), in remote ROI (as in D; 2.0% ± 3.5%, n = 11), and with 20 μM bumetanide whole-cell (−1.4% ± 3.3%, n = 9); ^∗^p < 0.05 (df = 15); ^∗∗∗^p < 0.005 (df = 23).

Although HFS in the bulk of tissue potentiates multiple synapses, memory trace formation is likely to involve changes at individual connections. We therefore set out to test how LTP at individual synapses affects PAPs. First, we modified an established protocol in which LTP at a CA3-CA1 synapse is induced by local glutamate spot-uncaging (Harvey and Svoboda, 2007; Matsuzaki et al., 2004; Yasuda et al., 2003). We held a CA1 pyramidal cell in voltage clamp and spot-uncaged glutamate (1-ms pulse) near its dendritic spine (Figure 4A) achieving a typical unitary EPSC (Figure 4B; STAR Methods). Next, we switched to current clamp maintaining V_m_ at −60 to −65 mV, the range for CA1 pyramids in freely moving animals (Epsztein et al., 2010). Here, applying the spot-uncaging sequence that mimics the HFS protocol generated postsynaptic depolarization sufficient to trigger strong Ca^2+^ entry reported by OGB-1 (Figures S4A and S4B). Switching back to voltage clamp revealed potentiation of single-pulse EPSCs (Figure 4B), which was induced robustly at every recorded synapse (7 out of 7 cells) (Figures 4B and 4C).

Because CA3-CA1 synapses are only ∼0.5 μm apart (Rusakov and Kullmann, 1998), spot-uncaging HFS should potentiate at least one synapse nearby, whether or not the unclamped postsynaptic cell is visualized. We therefore loaded an astrocyte with AF 594 and OGB-1 and applied spot-uncaging while monitoring VF and Ca^2+^ in the adjacent PAPs (Figure 4D). The HFS uncaging sequence in most cases evoked a local Ca^2+^ rise in PAPs (Figures 4D, 4E, and S4D), indicating robust glutamate release. In such cases, we detected PAP VF reduction near the spot (Figures 4F, 4G, and S4C–S4E; Video S2), but no changes either in remote ROIs (>3 μm away, Figure 4D) or without MNI-glutamate in the bath (Figures 4G and 4H). Unsurprisingly, the VF change was smaller than under bulk LTP induction (Figures 1, 2, and 3) where a co-operative effect was likely. Blocking NKCC1 with whole-cell loaded bumetanide suppressed the LTP-associated PAP change (Figure 4H). A complementary strategy, in which astrocytes were imaged using the membrane-bound GFP (AAV5.GfaABC1D.Pi.lck-GFP.SV40) produced a qualitatively identical result, with the PAP withdrawal lasting for up to 100–120 min post-induction (Figures S4F–S4H).

Video S2. Monitoring Local PAP VF during LTP Induction by Spot Uncaging of Glutamate, Related to Figure 4 Time-lapse series, example of a CA1 astrocyte loaded whole-cell with Alexa Fluor 594 (single optical section, λ_x_^2p^ = 810 nm). White circle, example ROI for PAP VF monitoring; see Figures 4D and 4E for further detail.

### LTP Induction Increases Glutamate Traveling Distance

**Figure 5:**
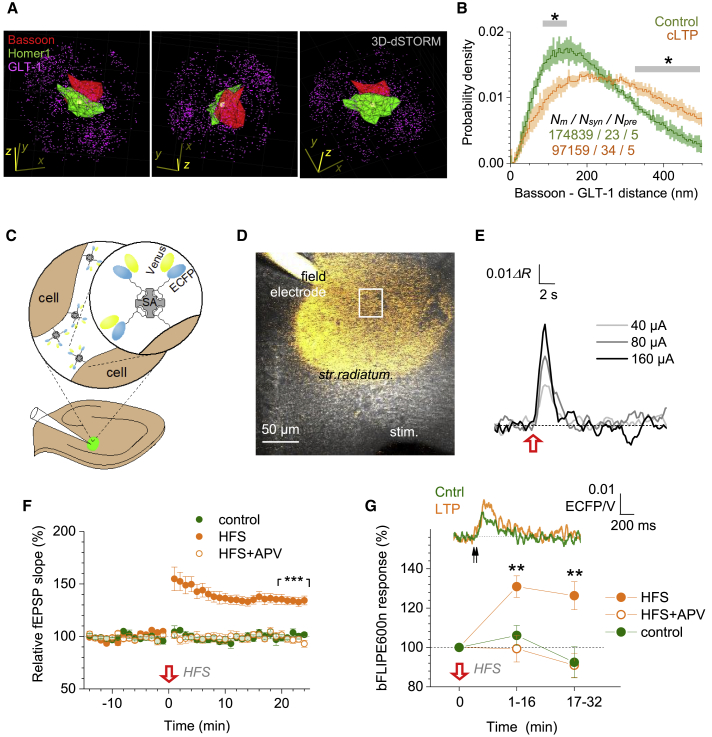
LTP Induction Triggers Withdrawal of Glial Glutamate Transporters Boosting Extracellular Glutamate Transient (A) Perisynaptic patterns of bassoon (red cluster), Homer 1 (green cluster), and GLT1 (magenta dots) molecules localized with 3D dSTORM; one-synapse example, three viewing angles shown; x-y-z scale bars, 500 nm (STAR Methods). (B) Nearest-neighbor distances (probability density, mean ± SEM) between GLT1 and bassoon, in control tissue and ~30 min after cLTP induction (Figures S5A and S5B; STAR Methods); sample size: *N*_*m*_, inter-molecular distances; *N*_*syn*_, synapses; *N*_*pre*_, slices; SEM relates to *N*_*pre*_ = 5; ^∗^p < 0.05 (gray segments, significant difference). (C) Diagram, extracellular immobilization of bFLIPE600n (Venus and ECFP attachments shown) via biotinylation and attachment to streptavidin (SA) (Figure S5D; STAR Methods) in *s. radiatum* (delivery pipette shown). (D) Experimental design: sensor-injecting pipette (field) records fEPSPs evoked by Schaffer collateral stimulation (stim) while bFLIPE600n signal is monitored within an adjacent ROI (rectangle). (E) Example, glutamate signal reported by bFLIPE600n (Δ*R*, ECFP/Venus signal ratio) in response to Schaffer collateral HFS (100 Hz for 1 s, red arrow; 10 μM NBQX, 50 μM D-APV) in *s. radiatum* (also Figures S5E and S5F). (F) Relative fEPSP slope (%, mean ± SEM) in control (green, n = 8 slices), during LTP induction (n = 14, orange), and with 50 μM APV present (n = 7, orange empty); ^∗∗∗^p < 0.005, difference over 20–25 min post-induction. (G) Traces, bFLIPE600n response to paired-pulses (20 Hz, arrows; mean ± SEM) in control (green) and ~25 min after LTP induction (orange). Plot, summary (notations as in F); ^∗∗^p < 0.01, difference between LTP and either control or APV datasets.

We next hypothesized that PAP withdrawal alters perisynaptic occurrence of GLT1. To test this, we turned to dSTORM, a super-resolution technique that we adapted previously (Heller et al., 2020), aiming to map 3D co-ordinates of the presynaptic protein bassoon, the PSD protein Homer1, and local GLT1 (Figure 5A). To potentiate synapses in bulk, we employed the classical chemically induced LTP (cLTP) protocol in acute hippocampal slices (Otmakhov et al., 2004) (Figure S5A).

Three-color dSTORM revealed 3D perisynaptic patterns of GLT1 molecules (Figures 5A and S5B). In potentiated slices, GLT1 occurred consistently further away from bassoon, compared to control (Figure 5B). Because bassoon is a key player in synaptic vesicle release (Gundelfinger et al., 2016), this suggested that glutamate released from potentiated synapses travels further, compared to control, to reach GLT1. We could not detect a similar trend for GLT1-Homer1 distances (Figure S5C), possibly because Homer1 showed a relatively dispersed pattern across the spine head.

### Induction of LTP Extends Extracellular Exposure of Released Glutamate

To test whether the withdrawal of GLT1-enriched PAPs indeed prompts increased extracellular travel of released glutamate, we employed the optical glutamate sensor FLIPE600n (Okumoto et al., 2005) immobilized in the extracellular space (Okubo et al., 2010), as described previously (Zhang et al., 2018) (Figures 5C and S5D; STAR Methods). The sensor showed high glutamate sensitivity (Figure S5E) and could be delivered with a patch-pipette (Figures 5C and 5D). Burst stimulation of Schaffer collaterals induced a robust optical response (Figures 5E and S5F), which was significantly increased after LTP induction (Figures 5F and 5G). Because in similar settings, LTP induction has no effect on the overall amount of released glutamate (Diamond et al., 1998; Lüscher et al., 1998), the increased bFLIPE600n response suggests a greater sensor exposure to the extrasynaptic glutamate transient. To test this at the synaptic level, we carried out two further experiments, as described below.

### LTP Induction Widens Spatial Extracellular Transients of Released Glutamate

In the first experiment, we expressed the glutamate sensor iGluSnFR (Marvin et al., 2013) in area CA1, in either neurons or astroglia of the mouse hippocampus (STAR Methods). Optical iGluSnFR response to paired-pulse stimuli faithfully reflected Ca^2+^-dependent changes in fEPSPs (Figures S6A and S6B) and also their preserved paired-pulse ratio after LTP induction (Figure S6C) (Diamond et al., 1998; Lüscher et al., 1998).

**Figure 6:**
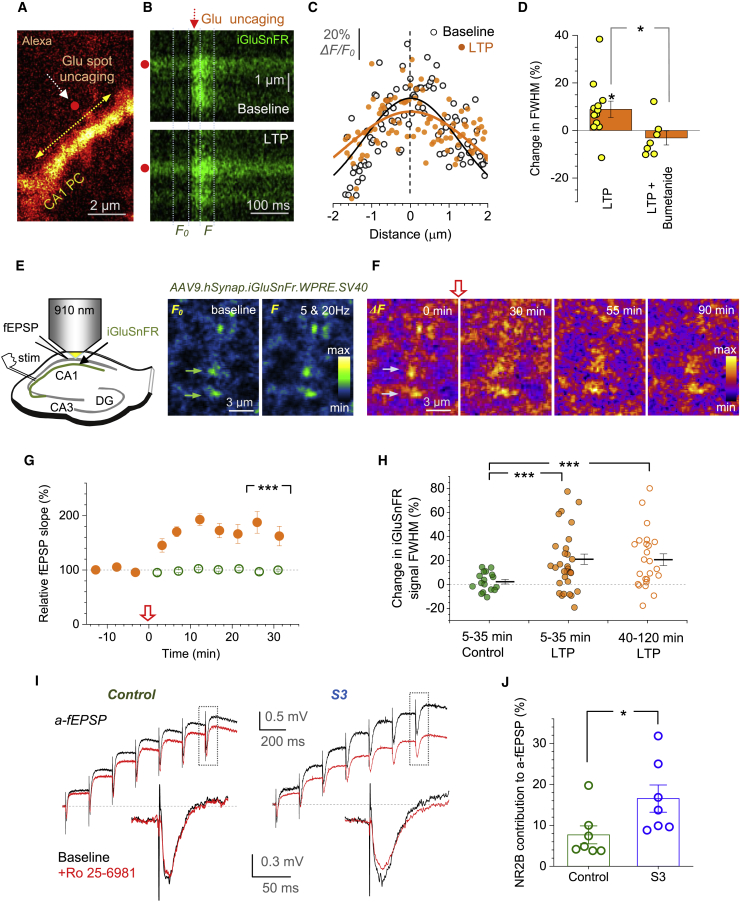
LTP Induction Broadens Evoked Extracellular Glutamate Transients (A) Dendritic fragment, CA1 pyramidal cell (AF 594 channel); red dot, glutamate uncaging spot; yellow arrow, line scan position for iGluSnFR monitoring (Figures S6A–S6C). (B) Line scans (as in A; iGluSnFR channel) showing fluorescence transients in response to a 1 ms uncaging pulse (arrow, onset; red dot, position), before (top) and 20–25 min after the spot-uncaging LTP induction (bottom); dotted lines, time windows to sample baseline (*F*_*0*_) and evoked (*F*) fluorescence profiles, giving signal profile Δ*F* = *F* − *F*_*0*_ (STAR Methods). (C) iGluSnFR fluorescence profiles (dots, pixel values) from test in (B); zero, uncaging spot position; black and orange lines, best-fit Gaussian. (D) Summary of tests shown in (A)–(C): relative change (%, mean ± SEM) in Δ*F*/*F*_*0*_ signal full-width-at-half-magnitude (FWHM) ~25 min after LTP induction (LTP, 9.0% ± 3.4%; n = 12; ^∗^p < 0.03), and with 20 μM bumetanide inside astroglia (LTP+Bumetanide; −3.1% ± 3.0%; n = 7; ^∗^p < 0.02, df = 15; Figures S6D–S6G); dots, individual tests. (E) Diagram, monitoring evoked glutamate release from Schaffer collateral boutons with iGluSnFR, acute slices. Images: iGluSnFR fluorescence landscape *s. radiatum* in resting conditions (*F*_*0*_) and during five stimuli at 20 Hz (*F*); arrows, two tentative axonal boutons, false colors. (F) Evoked iGluSnFR signal landscapes (Δ*F* = *F* − *F*_*0*_; ROI as in E) just before (0 min, as in E) and 30, 55, and 90 min after LTP induction (red arrow; Figure S6H; STAR Methods); false colors. (G) Relative fEPSP slope (%, mean ± SEM, n = 8 slices), protocol as in (E) and (F); arrow, LTP induction; ^∗∗∗^p < 0.001 (relative to no-HFS control, n = 4; over 25–35 min post-induction; df = 10). (H) The FWHM of evoked iGluSnFR Δ*F* signals relative to baseline, over 5–35 min in control conditions (control, n = 17 boutons), 5–35 min (n = 31), and 40–120 min (n = 21) after LTP induction, as shown; dots, individual boutons; bars, mean ± SEM; ^∗∗∗^p < 0.005 (df = 46; 4 slices). (I) Upper traces, examples of CA1 astrocyte-recorded fEPSPs (a-fEPSP, current clamp, isolated NMDAR component; 3–5 trial average) evoked by 7 stimuli at 5 Hz, in baseline conditions (black) and after blocking GluN2B-containing NMDARs (1 μM Ro 25-6981, red); control cell and one dialyzed with 200 μM peptide S3 shown, as indicated; lower traces, fragments (rectangles) showing the 7th a-fEPSPs (pre-pulse baseline adjusted; see Figure S6I for extended traces). (J) Summary of tests shown in I; ordinate, reduction of the a-fEPSP amplitude by Ro 25-698; dots, individual cells; bars, mean ± SEM; ^∗^p < 0.05 (n = 7 in control and S3; df = 12).

We next monitored the spatial spread (FWHM) of the iGluSnFR response to a 1-ms glutamate spot-uncaging pulse, either near a postsynaptic dendrite (Figure 6A) or within an astrocyte ROI (Figure S6A) using line scans (Figures 6B and S6F), before and 10–30 min after the spot-uncaging LTP protocol (as in Figures 4A and 4B). LTP induction appeared to widen the iGluSnFR signal (Figures 6C and 6D; n = 12) but not when the iGluSnFR-expressing astrocyte was dialyzed with bumetanide to block NKCC1 (Figures 6D, S6F, and S6G).

The second test aimed at detecting PAP changes near active axonal boutons visualized using relatively sparse iGluSnFR expression in *S. radiatum*. We focused on boutons that responded optically to Schaffer collateral stimulation (five pulses at 20 Hz; Figure 6E) and recorded iGluSnFR signal landscapes before and up to 90–120 min after LTP induction (Figures 6F and S6H). Again, LTP induction increased the signal FWHM, for up to 120 min (Figure 6H), although some boutons showed no change (Figure 6H), probably reflecting non-potentiated connections. The average effect was larger than that under spot-uncaging (Figure 6D), likely because burst stimulation amplifies glutamate spillover (Lozovaya et al., 1999).

### Cofilin-Dependent PAP Shrinkage Boosts Activation of Extrasynaptic NMDARs

Because astrocyte dialysis with peptide S3 reduces PAP VF in baseline conditions (Figures 3G and 3H), we asked if this would, on its own, boost glutamate spillover. We noted that extrasynaptic NMDARs are predominantly GluN2B subunit-containing so that their contribution to NMDAR EPSCs/EPSPs in area CA1 varies with the extent of glutamate spillover, in particular during stimulus bursts (Lozovaya et al., 2004; Papouin et al., 2012; Scimemi et al., 2004). To confine ourselves to one astrocyte and its “territorial” synapses, we recorded local fEPSPs (7 pulses at 5 Hz) through the astrocyte patch pipette, previously termed as a-fEPSPs (Henneberger et al., 2010; Henneberger and Rusakov, 2012). In baseline conditions, blocking GluN2B-containing NMDARs with Ro 25-6981 unveiled their 8% ± 2% (n = 7) contribution to the 7th NMDAR a-fEPSP. However, if the astrocyte was dialyzed with S3, this contribution was 17% ± 3% (n = 7) (Figures 6I, 6J, and S6I). Thus, withdrawal of PAPs per se was capable of boosting glutamate escape.

### Whisker-Stimulation-Induced LTP Reduces PAP Presence Near Firing Axons

To assess physiological relevance of our observations, we turned to tests *in vivo*. We focused on the established protocol of LTP induced at the thalamocortical synapses in the barrel cortex (layer II/III) by contralateral rhythmic whisker stimulation (RWS) (Gambino et al., 2014; Mégevand et al., 2009).

**Figure 7:**
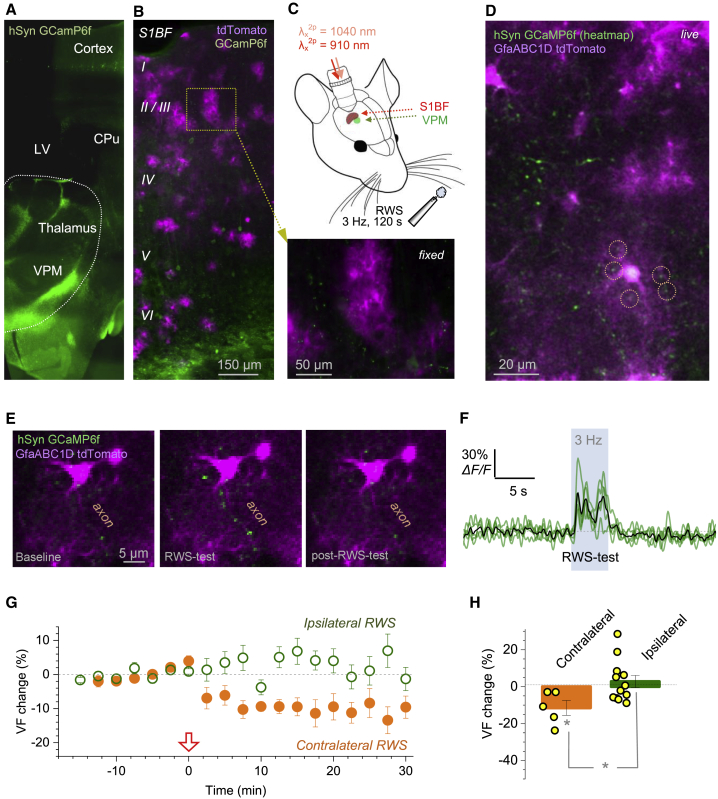
Whisker-Stimulation LTP Protocol in the Barrel Cortex *In Vivo* Triggers PAP VF Reduction in Astroglia Trespassed by Stimulated Axons (A) Expression of GCaMP6f 3 weeks post-transfection (STAR Methods) into the mouse ventral posteromedial nucleus (VPM), coronal section; LV, lateral ventricle; CPu caudate putamen; wide-field image, fixed tissue. (B) Composite post hoc image, barrel cortex area (coronal section), with astroglia expressing GfaABC1D tdTomato (magenta; STAR Methods) and neuronal structures expressing GCaMPf6 (green); dotted rectangle (inset, arrow) highlights astrocytes with axonal boutons occurring nearby. (C) Experiment diagram: 2PE imaging of the barrel cortex (S1BF) through a cranial window, with two fs lasers. LTP induction protocol uses RWS (5 Hz air-puffs for 120 s) on the contralateral side. (D) Live barrel cortex view (S1BF) through the cranial window (λ_x_^2^^P^ = 1,040 and 910 nm, single focal section). Green (GCaMPf6), heatmap of axonal signals firing in response to RWS; magenta (tdTomato), local astroglia; circles, examples of ROIs for PAP VF readout in proximity to RWS-responding thalamocortical axons (green; Figures S7A and S7B). (E) Example, a thalamocortical axon in S1BF (GCaMP6f, green) crossing astroglial territory (tdTomato, magenta), with boutons responding to an RWS test (3 Hz, 5 s) with Ca^2+^ elevations (middle panel). (F) Time course of Ca^2+^ signal (GCaMP6f) at five axonal boutons (green traces) shown in (E); black line, average. (G) PAP VF change (%, mean ± SEM), during RWS LTP induction protocol (arrow, onset), near axonal boutons responding to contralateral RWS (orange, n = 5 cells, 3 animals), and in during ipsilateral RWS (n = 12 cells, 4 animals). (H) Summary of experiments in (G): PAP VF change (%, mean ± SEM) over 15–30 min after the RWS LTP protocol onset; dots, data from individual cells; ^∗^p < 0.04 (t test, df = 15 for two-sample comparison).

Building upon our previous *in vivo* imaging protocols (Reynolds et al., 2019; Savtchenko et al., 2018; Zheng et al., 2015), we expressed the green Ca^2+^ indicator GCaMP6f in the ventral posteromedial nucleus (VPM) that sends axonal projections to the barrel cortex (Figure 7A). In parallel, we sparsely expressed the red-shifted, cytosol-soluble indicator (GfaABC1D) tdTomato in the barrel cortex astroglia (Figure 7B). Thus, we could monitor, through an implanted cranial window, fine astroglial morphology together with presynaptic Ca^2+^ dynamics in individual thalamocortical projections (Figures 7C and 7D).

First, we confirmed that PAP VF readout with tdTomato was similar to that with AF 594 (Figures S7A and S7B). Next, within the tdTomato-expressing astrocyte domains, we found axonal boutons that showed Ca^2+^ elevations in response to an RWS test (3 Hz air puffs for 5 s) (Figures 7E and 7F). This enabled us to monitor PAP VF in within ∼3 μm of active boutons, before and after LTP induction by RWS (3 Hz air 100 ms stimuli for 120 s) (Figure 7C). LTP induction by contralateral RWS triggered PAP VF reduction (5 cells, 3 animals) whereas the same protocol applied ipsilaterally had no effect (12 cells, 4 animals) (Figures 7G and 7H).

We used a similar imaging design in a complementary test in acute hippocampal slices. We loaded a CA3 pyramidal cell with OGB-1 and traced its axon into area CA1, which was populated with tdTomato-expressing astroglia (Figures S7C and S7D). We then paired presynaptic spikes (triggered by somatic depolarization pulses) with postsynaptic CA1 pyramidal cell depolarization induced by periodic extracellular stimuli (Figures S7E and S7F). This LTP-inducting pairing protocol reduced PAP VF near activated axonal boutons by 12% ± 2% (n = 5) whereas no such reduction occurred away from the firing axon (change 3.4% ± 1%, n = 10; difference at p < 0.01, degrees of freedom [df] = 13) (Figure S7E; Video S3).

Video S3. Monitoring Local PAP VF Near Active Axonal Boutons during LTP Induction by Pre/Postsynaptic Pairing, Related to Figure S7 Time-lapse series, multiplexed imaging of CA1 astrocytes expressing tdTomato (magenta, single optical section), which territories are crossed by a single CA3 pyramidal cell axon (whole-cell loaded with OGB-1, green); Circles 1-4, example test ROIs for PAP VF near active axons; circle 5, somatic ROI; circle 6, example control ROI; see Figures S7C–S7G for further detail.

### LTP Induction Prompts NMDAR-Mediated Cross-Talk among Synapses

To test if the LTP-associated increase in glutamate escape promotes activation of high-affinity NMDARs at neighboring, non-active connections, we used a protocol established specifically to monitor NMDAR-mediated cross-talk among independent CA3-CA1 synapses (Scimemi et al., 2004). It takes advantage of the use-dependent NMDAR inhibitor MK801, which blocks the receptor channel upon its opening. Thus, if NMDARs at non-active (silent) synapses get blocked by MK801 they must have been activated by glutamate molecules escaping from nearby active synapses.

**Figure 8:**
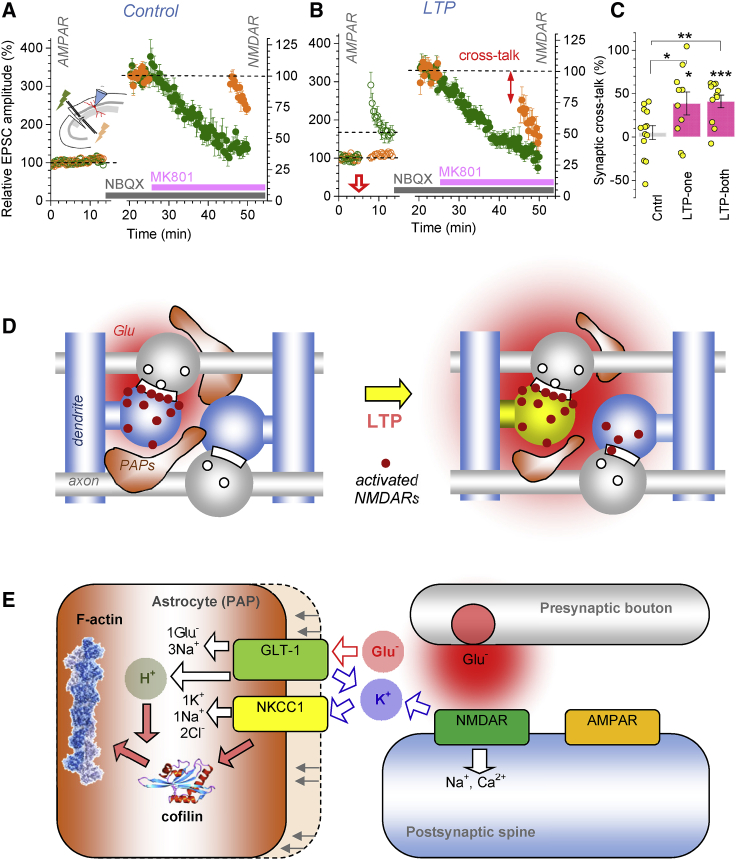
LTP Induction Boosts NMDAR-Mediated Inter-synaptic Cross-Talk (A) Inset diagram, experiment design to test NMDAR-mediated cross-talk between two afferent pathways (green and orange lightning) (Scimemi et al., 2004) (Figure S8A; STAR Methods). Plot, relative EPSC amplitude (mean ± SEM, n = 13), with single stimuli, 20 s apart, applied alternately to the two pathways (green and orange). AMPAR EPSCs are recorded for 12–15 min (V_m_ = −70 mV; left ordinate), then NMDAR EPSCs for ~5 min (10 μM NBQX, V_m_ = −20 mV; right ordinate). Once MK801 is added, NMDAR EPSCs are recorded in active (green) pathway only. Resuming stimulation in the silent (orange) pathway reveals little change in the NMDAR EPSC amplitude compared to baseline (dotted line). (B) Experiment as in (A) but with LTP induced in the active pathway (red arrow; n = 7). Reduced NMDAR EPSCs in the silent (orange) pathway upon resumed stimulation (arrow, cross-talk) point to NMDAR activation by glutamate escaping from the active (green) pathway. (C) Summary of experiments in (A) and (B). The degree of cross-talk (percentage of one-pathway NMDARs activated by glutamate discharges at the other pathway; mean ± SEM), in control (Cntrl, n = 13), with LTP induced either in one (LTP-one, n = 10) or both (LTP-both, n = 11; Figures S8C and S8D) pathways, prior to NMDAR EPSC recordings; ^∗^p *<* 0.05 (df = 21 for Cntrl versus LTP-one), ^∗∗^p *<* 0.01 (df = 22), ^∗∗∗^p *<* 0.005. (D) Proposed changes in PAPs after LTP induction. In baseline conditions (left), PAPs restrict glutamate action to the synaptic cleft and some extrasynaptic NMDARs (red dots). After LTP induction (right), some PAPs withdraw, widening the pool of activated extrasynaptic NMDARs, including neighboring synapses. (E) Diagram, candidate cellular mechanisms of LTP-driven PAP withdrawal. LTP induction activates postsynaptic NMDARs and engages GLT1 transporters. This generates an extracellular K^+^ hotspot, activating the NKCC1-cofilin-1 pathway that engages, in a pH-sensitive manner, actin polymerization responsible for morphogenesis.

First, we used paired-pulse stimuli to confirm independence of two Schaffer collateral pathways converging to a CA1 pyramidal cell (Figure S8A). Second, we recorded AMPA receptor-mediated EPSCs (AMPAR EPSCs), then NMDAR EPSCs, elicited in either pathway (Figure 8A). Third, we applied MK801 and recorded declining NMDAR EPSC responses in one (active) pathway only (Figure 8A). When stimulation resumed in the other, silent pathway, its NMDAR EPSC amplitude was close to its baseline value (Figures 8A, top dotted line, and S8B, no-LTP, silent). Thus, the silent pathway had little cross-activation of its NMDARs by synaptic discharges in the active pathway.

The outcome was different when we induced LTP of AMPAR EPSCs in the active pathway (Figure 8B). Here, resuming stimulation of the silent pathway revealed reduced NMDAR EPSCs (Figure 8B, cross-talk). Thus, a proportion of NMDARs here must have been activated by glutamate escaping from synapses in the active pathway (see Discussion for quantitative estimates). LTP induction in the silent pathway, or in both pathways, produced similar outcome (Figures 8C and S8C). We confirmed that the trial-to-trial time decay of NMDAR EPSCs was similar among potentiated and non-potentiated pathways, suggesting no effects of LTP induction on the overall release probability (Figure S8D), as reported here (Figures S6A–S6C) and earlier (Diamond et al., 1998; Lüscher et al., 1998; Manabe and Nicoll, 1994).

## Discussion

### Biophysical Plausibility

Our results suggest that LTP induction prompts nanoscopic withdrawal of PAPs, which boosts extrasynaptic glutamate escape, thus enhancing NMDAR activation away from the release site, potentially at nearby synapses (Figure 8D). To assess biophysical plausibility of these events, we modeled CA3-CA1 synaptic environment (Figure S8E) (Zheng et al., 2008) and simulated three scenarios that might reflect our observations: GLT1-enriched PAPs (1) withdraw without losing any GLT1, (2) withdraw while losing some GLT1, or (3) re-arrange laterally with the same GLT1 numbers (Figure S8F), which partly exposes extrasynaptic NMDARs. After multiple runs (example in Video S4), scenario (1) appeared most likely in boosting remote NMDAR activation (Figure S8G).

Video S4. Monte Carlo Model of Extrasynaptic Glutamate Escape, NMDA Receptor, and Glutamate Transporter Activation, Related to Figure S8 Characteristic model run, front view of the synaptic cleft and nearby astroglial environment; further detail and notations in Figures S8E and S8F.

### Cellular Mechanisms of LTP-Dependent PAP Withdrawal

We found that the LTP-associated PAP withdrawal depends on NKCC1, a key morphology regulator in brain cell migration (Garzon-Muvdi et al., 2012; Haas and Sontheimer, 2010). In glioma cells, NKCC1 mediates dramatic hydrodynamic volume changes that enable invasion of intact tissue (Watkins and Sontheimer, 2011), probably by boosting intracellular chloride up to 140 mM (Habela et al., 2009). The NKCC1-regulated phosphorylation of cofilin-1 has been revealed in glioblastoma (Schiapparelli et al., 2017), and we found that inhibiting cofilin-1 phosphorylation with peptide S3 occluded LTP-induced PAP shrinkage, suggesting a shared molecular pathway. An alternative interpretation is that both mechanisms simply reduce PAP VF to a maximal degree. Yet, astrocyte dialysis with S3 does boost glutamate spillover, similar to the LTP case.

What activates NKCC1 upon LTP induction remains to be ascertained. One possibility is that intense activation of local NMDARs and GLT1 leads to a hotspot of K^+^ efflux (Shih et al., 2013). Classically, NKCC1 is activated by excess of external K^+^ (Russell, 2000) whereas proton transport by GLT1 could help boost cofilin-dependent actin assembly. Although this appears plausible (Figure 8E), a better understanding of the mechanisms relating PAP plasticity to NKCC1 and cofilin, and probably to other morphogenic agents of astroglia such as neuroligins (Stogsdill et al., 2017), requires a separate study.

### 3D EM: Faithful Representation of Live Tissue?

The relevance of fixed-tissue EM has recently been questioned: chemical fixation *in vivo* can cause linear tissue contraction by ∼18% resulting in ∼2% VF for the extracellular space and distorted PAP morphology (Korogod et al., 2015). However, different fixation protocols produce different outcomes. Here, we used rapid slice fixation by submersion: our earlier studies reported 5%–6% linear hippocampal shrinkage under a similar protocol (Rusakov et al., 1998), whereas *in vivo* fixation gave ∼12% extracellular space VF in area CA1 (Rusakov and Kullmann, 1998). In chemically fixed CA1 tissue, PAPs occupied ∼9% of tissue volume (Lehre and Rusakov, 2002), which falls within the range estimated here with live 2PE imaging. A similar correspondence was observed in other studies (Medvedev et al., 2014; Savtchenko et al., 2018).

We made no attempt to assess PAP shapes or exact position, which, in addition to protocol differences, might explain an apparent discrepancy with some previous results. For instance, smaller PAPs that occur closer to synapses might well count as an increased PAP occurrence (Lushnikova et al., 2009; Wenzel et al., 1991) even though their overall VF decreases. Similarly, we do not dispute previous findings reporting high PAP mobility or morphological plasticity detected with fluorescence imaging (Bernardinelli et al., 2014; Haber et al., 2006; Hirrlinger et al., 2004; Perez-Alvarez et al., 2014) but note that mobility of the fluorescent label, or fluctuations in focus, laser power, or tissue optical properties, might add to the perceived motility.

### PAP Withdrawal and Extrasynaptic Glutamate Actions

Remodeling of GLT1-enriched PAPs on the nanoscale will not affect total glutamate uptake by astroglia because all released molecules will still be bound by local GLT1 and taken up by the same astrocyte. Thus, LTP induction should have little effect on the astrocyte uptake currents measured by a somatic pipette (Diamond et al., 1998; Lüscher et al., 1998). However, reduced PAP coverage suggests that glutamate should dwell longer and travel further in the extracellular space, thus allowing high-affinity optical sensors to compete more successfully with GLT1 (Armbruster et al., 2020; Kopach et al., 2020). Thus, the optical glutamate signal is enhanced after LTP induction.

We examined NMDAR-mediated cross-talk between two independent pools of CA3-CA1 synapses and found that, following LTP induction, ∼120 discharges in the active pool activated ∼40% NMDARs in the silent pool. Although this suggests only ∼0.4% per discharge, this protocol activates only 2%–3% of CA3-CA1 connections (Scimemi et al., 2004). With the synaptic nearest-neighbor distance in CA1 of ∼0.5 μm (Rusakov and Kullmann, 1998), 2% synapses will be separated by 0.5 × (0.02^−1/3^) ∼1.8 μm. The travel distance increase from 0.5 to 1.8 μm corresponds to a >100-fold drop in the glutamate concentration transient post-release (Rusakov, 2001; Zheng et al., 2008). Thus, cross-talk among 2%–3% synapses accumulated over ∼120 discharges could underestimate cross-talk between nearest neighbors per discharge.

The increased exposure of glutamate to the extracellular space after LTP induction might explain why some earlier studies reported increased extracellular glutamate transients detected with micro-dialysis (Bliss et al., 1986; Errington et al., 2003). It might also explain the reduced NMDAR EPSC variability at CA3-CA1 synapses (Kullmann et al., 1996), an enhanced local excitability of pyramidal cell dendrites (Frick et al., 2004), and why LTP at one synapse could lower the LTP induction threshold at its neighbors (Harvey and Svoboda, 2007). Other important consequences could be a boost in NMDAR-driven dendritic spikes (Chalifoux and Carter, 2011), facilitated plasticity at silent connections nearby (Tsvetkov et al., 2004), or increased heterosynaptic depression (Vogt and Nicoll, 1999).

### PAP Remodeling on Longer Timescales

Our observations in slices were necessarily limited to 30–90 min after LTP induction, and to 30–35 min *in vivo* (to avoid concomitants of animal stress in 2- to 3-h long experiments). This does not preclude the possibility for PAP coverage to re-establish itself on a longer timescale. Indeed, unlimited accumulation of LTP must lead to runaway excitation unless synaptic weight re-scaling follows it. One might therefore expect a similar dynamic sequence of PAP remodeling on a longer timescale, which remains an important and intriguing question to be addressed in chronic experiments.

## STAR★Methods

### Key Resources Table

**Table:** 

REAGENT or RESOURCE	SOURCE	IDENTIFIER
**Antibodies**		

Mouse monoclonal (SAP7F407) anti-bassoon	Novus Biologicals	Cat. # NB120-13249; RRID: AB_788125
Rabbit polyclonal anti-Homer1	Synaptic Systems	Cat. #1 60003; RRID: AB_887730
Guinea pig polyclonal anti-GLT-1	Merck	Cat. # AB1783; RRID: AB_90949
Donkey anti-mouse IgG	Biotium	Cat. # 20105; RRID: AB_10557030
Goat anti-rabbit IgG	Rockland	Cat. #6 11-152-122S; RRID: AB_10893832
Donkey anti-guinea pig IgG	Jackson ImmunoResearch Labs	Cat. # 706-606-148; RRID: AB_2340477

**Bacterial and Virus Strains**		

AAV9.hSynap.iGluSnFr.WPRE.SV40	Penn Vector Core; Marvin et al., 2013	Addgene Cat. # 98929-AAV9
AAV5.GfaABC1D.Pi.lck-GFP.SV40	Penn Vector Core	Addgene Cat. #105598-AAV5
AAV5.GfaABC1D.Pi.lck-GCaMP6f.SV40	Penn Vector Core	Addgene Cat. # 52924-AAV5 Batch: CS0846L
AAV GFAP-iGluSnFR	Penn Vector Core	Addgene Cat. # 44332-AAV5
AAV5.GfaABC1D.cyto-tdTomato.SV40	Penn Vector Core; Shigetomi et al., 2013	Cat:AV-5-PV3106; Lot: V5606L; RRID: Addgene_44332
AAV9.Syn.GCaMP6f.WPRE.SV40	Penn Vector Core	Cat: AV-9-PV2822; Lot: CS0932; RRID: Addgene_100837

**Chemicals, Peptides, and Recombinant Proteins**		

DNI-GLU-TFA	Femtonics	DNI-GLU-TFA
Picrotoxin	Tocris	Cat. # 1128
CGP 52432	Tocris	Cat. # 1246/10
Bumetanide	Tocris	Cat. # 3108
Biocytin	Sigma-Aldrich	Cat. # B4261
NMDA	Tocris	Cat. # 0114/50
Biotin-tag, Synthetized as double stranded DNA	Epoch Life Science	PinPoint™ Xa-1 (Promega #V2031)
pRSET FLIPE-600n	Wolf B. Frommer	Addgene #13537
pRSET bFLIPE600n	This paper	N/A
Sulfo-NHS EZ Link Biotin	ThermoFisher	Cat. #21217
Streptavidin, unconjugated	ThermoFisher	Cat. #SNN1001
(+)-MK-801 hydrogen maleate	Sigma Aldrich	Cat. #M107
D-APV	Abcam	Cat. #ab120003
NBQX disodium salt	Abcam	Cat. #ab120046
Ro 25-6981 maleate salt	Abcam	Cat. #ab120290
S3 Fragment, ADF/cofilin	Anaspec	Cat. #AS-62637
Oregon Green 488 BAPTA-1	ThermoFisher	Cat. #O6806
Picrotoxin	Tocris	Cat. #1128
CGP52432	Tocris	Cat. #1246
MNI-caged-L-glutamate	Bio-Techne Ltd	Cat-#1490
DL-AP5	Bio-Techne Ltd	Cat. # 0105
CGP 52432	Bio-Techne Ltd	Cat. # 1246
NBQX disodium salt	Bio-Techne Ltd	Cat. # 1044
TFB-TBOA	Bio-Techne Ltd	Cat. # 2532
Tetrodotoxin citrate	Bio-Techne Ltd	Cat. # 1069
WIN 55,212-2 mesylate	Bio-Techne Ltd	Cat. # 1038
DHPG	Bio-Techne Ltd	Cat. # 0805
Muscimol	Bio-Techne Ltd	Cat. # 0289
MK 801	Bio-Techne Ltd	Cat. # 0924
Chonditinase ABC	Sigma-Aldrich	Cat-# C3667
nEphrin-A4/FC Chimera	Sigma-Aldrich	Cat. # E0403
Fc Fragment	Sigma-Aldrich	Cat. # AG714
TGN-020	Sigma-Aldrich	Cat. # SML0136
Bumetanide	Sigma-Aldrich	Cat. # B3023
Alexa Fluor-594 Hydrazide	ThermoFisher Scientific	Cat.# A10438
Alexa Fluor-488 Hydrazide	ThermoFisher Scientific	Cat.#A10436
Fluo-4, Pentapotassium Salt	ThermoFisher Scientific	Cat.# F14200
Oregon Green 488 BAPTA-1, Hexapotassium Salt	ThermoFisher Scientific	Cat.# O6806
NPE-Caged Ins 1,4,5-P_3_	ThermoFisher Scientific	Cat. # I23580
Antisedan	Vetoquinol	Cat. # 459180
Carprofen Rimadyl	Zoetis	N/A
Forskolin	Sigma-Aldrich	Cat. # F6886/3917
VECTASTAIN® ABC-HRP Kit	Vector labs, USA	Cat. #PK-4000
Picrotoxin	Sigma-Aldrich	Cat. #P1675; CAS: 124-87-8
Forskolin	Cayman Chemical	Cat. #11018; CAS: 66575-29-9
Rolipram	Cayman Chemical	Cat. #31111; CAS: 85416-75-7
NaBH_4_	Sigma-Aldrich	Cat. #71320; CAS: 16940-66-2
PBS	Sigma-Aldrich	Cat. #4417
CuSO_4_	Sigma-Aldrich	Cat. #C8027; CAS: 7758-99-8
NH_4_Cl	Sigma-Aldrich	Cat. #254134; CAS: 12125-02-9
Saponin	Bio Basic	Cat. #SB4521; CAS: 8047-15-2
BSA	Sigma-Aldrich	Cat. #A7906 CAS: 9048-46-8
Urea	Sigma-Aldrich	Cat. #U6504; CAS: 57-13-6
Glycerol	Fisher Scientific	Cat. #BP229-1 CAS: 56-81-5
Triton X-100	Sigma-Aldrich	Cat. #T9284; CAS: 9002-93-1
Agarose	Sigma-Aldrich	Cat. #9539; CAS: 9012-36-6
Catalase	Sigma-Aldrich	Cat. #C40; CAS: 9001-05-2
glucose oxidase	Sigma-Aldrich	Cat. #G2133; CAS: 9001-37-0
glucose	Sigma-Aldrich	Cat. #G8270; CAS: 50-99-7
KCl	Sigma-Aldrich	Cat. #P9333; CAS: 7447-40-7
MEA-HCl	Sigma-Aldrich	Cat. #M6500; CAS: 56-57-0
PFA	Sigma-Aldrich	Cat. #P6148; CAS: 30525-89-4
TCEP	Sigma-Aldrich	Cat. #C4706; CAS: 51805-45-9
Tris base	Sigma-Aldrich	Cat. #33742; CAS: 77-86-1
DAB	Sigma-Aldrich	Cat. #D5905-50TAB

**Experimental Models: Organisms/Strains**		

Mouse: C57BL/6J	Charles River Laboratories	C57BL/6NCrl Strain: 0159
Mouse: C57BL/6J	Charles River UK	RRID: IMSR_JAX:000664
Rat: Sprague-Dawley	Charles River Laboratories	Crl:CD (SD) Strain: 0204
Transgenic mouse line hGFAP-EGFP	Frank Kirchoff; Nolte et al., 2001	MGI ID:6188855
Wistar rats	Charles River Laboratories	Strain Code: 003
Mouse: *Aqp4*^*flox/flox*^*.* Strain background: C57BL/6J	Ole Petter Ottersen; Haj-Yasein et al., 2011	N/A
Thy1-YFP-H: B6.Cg-Tg(Thy1-YFPH)2Jrs/J	The Jackson Laboratory	Stock No: 003782

**Software and Algorithms**		

ImageJ	NIH	RRID:SCR_003070; https://imagej.nih.gov/ij/
pClamp10	Molecular Devices	RRID: SCR_011323; https://www.moleculardevices.com/products/axon-patch-clamp-system/acquisition-and-analysis-software/pclamp-software-suite
OriginPro	OriginLab Inc	RRID: SCR_014212; https://www.originlab.com/origin
MES 4.x-5.x	Femtonics Ltd.	RRID:SCR_018309; https://uk.mathworks.com/products/connections/product_detail/femtonics-mes.html
Imspector Image Acquisition & Analysis Software v0.1	Abberior Instruments Development Team	RRID:SCR_015249; https://imspectordocs.readthedocs.io/en/latest/intro.html
Huygens STED Deconvolution Software	Huygens Professional (SVI)	RRID:SCR_014237; https://svi.nl/Huygens-Deconvolution
WinWCP Versions 4.x-5.x	Strathclyde Electrophysiology Software	RRID:SCR_014270; http://spider.science.strath.ac.uk/sipbs/software_ses.htm
SymPhoTime 64	PicoQuant	RRID:SCR_016263; https://www.picoquant.com/products/category/software/symphotime-64-fluorescence-lifetime-imaging-and-correlation-software
SEM. Align 1.26b	John Fiala, Boston University	https://synapseweb.clm.utexas.edu
Trace 1.26b	John Fiala, Boston University	https://synapseweb.clm.utexas.edu
Autodesk 3D studio Max 8	Autodesk	https://www.autodesk.com
MATLAB	Mathworks	RRID:SCR_001622; https://uk.mathworks.com/products/matlab.html

**Other**		

Multiclamp 700B	Molecular Devices	RRID:SCR_018455
Olympus FluoView1000	Olympus	RRID:SCR_014215
Femto2D	Femtonics	Femto2D
Femto3D RC	Femtonics	Femto3D RC
BioRad Radiance 2100	BioRad	Radiance 2100

### Resource Availability

#### Lead Contact

Further information and requests for resources and reagents should be directed to and will be fulfilled by the Lead Contact, Dmitri Rusakov (d.rusakov@ucl.ac.uk)

#### Materials Availability

This study did not generate new unique reagents.

#### Data and Code Availability

The data supporting the current study have not yet been deposited because their highly diverse nature and formats make it impractical but they are fully available from the corresponding author on request. Original or source data for figures in the paper are also available on request.

### Experimental Model and Subject Details

#### Animals

All animal procedures were conducted in accordance with the European Commission Directive (86/609/ EEC), the United Kingdom Home Office (Scientific Procedures) Act (1986), and all relevant national (France, Germany) and institutional guidelines. Details on each of the animal models employed are given throughout the text and summarized below. All animals were maintained in controlled environments as mandated by national guidelines, on 12hr light/dark cycles, with food and water provided *ab libitum*.

For *ex vivo* electrophysiology and imaging, a combination of Wistar rats (3 – 5 weeks old, male), Sprague-Dawley rats (3 - 5 weeks old, male), knockout (KO) and transgenic mice (3 – 5 weeks old, male) were employed, in separate experimental designs as indicated. For experiments requiring viral-mediated expression of optical sensors, male and female wild-type C57BL/6 mice (Charles River Laboratories) were injected at 3 - 4 weeks of age with viral vectors and acute slices were obtained 2 - 4 weeks later. hGFAP-EGFP mice (Nolte et al., 2001) were kindly supplied by Frank Kirchhoff. AQP4 KO mice (Haj-Yasein et al., 2011) were backcrossed with C57BL/6 mice for five generations before intercrossing to yield KO (−/−) and wild-type (+/+) mice. For STED microscopy, organotypic hippocampal slice cultures were prepared from 5 - 7 day old Thy1-YFP mice (Jackson Laboratory).

For *in vivo* recordings, group-housed male and female wild-type C57BL/6 mice (Charles River Laboratories) were used. Animals served as their own controls through the use of ipsi- and contralateral stimuli as specified below. All animals were injected with viral vectors at 3 – 4 weeks, and cranial windows were implanted 2 weeks later. Imaging was performed at between 6 and 12 weeks of age, at least 3 weeks after injection of viral vectors.

### Method Details

#### Preparation of acute slices

350 μm thick acute hippocampal slices were obtained from three- to five-week-old male Sprague-Dawley or Wistar rats, or, alternatively, from wild-type, knockout, and transgenic mice, as explained in the text and detailed below. Slices were prepared in an ice-cold slicing solution containing (in mM): NaCl 75, sucrose 80, KCl 2.5, MgCl_2_ 7, NaH_2_PO_4_ 1.25, CaCl_2_ 0.5, NaHCO_3_ 26, ascorbic acid 1.3, sodium pyruvate 3, and glucose 6 (osmolarity 300-305), stored in the slicing solution at 34°C for 15 minutes before being transferred to an interface chamber for storage in an extracellular solution containing (in mM): NaCl 126, KCl 2.5, MgSO_4_ 1.3, NaH_2_PO_4_ 1, NaHCO_3_ 26, CaCl_2_ 2, and glucose 10 (pH 7.4, osmolarity adjusted to 295-305). All solutions were continuously bubbled with 95% O_2_/ 5% CO_2_. Slices were allowed to rest for at least 60 minutes before recordings started. For recordings, slices were transferred to the submersion-type recording chamber and superfused, at 33-35°C unless shown otherwise. Where required, 50-100 μM picrotoxin and 5 μM CGP52432 were added to block GABA receptors and a cut between CA3 and CA1 was made to suppress epileptiform activity.

#### Electrophysiology *ex vivo*

Electrophysiological examination of astrocytes was carried out as previously described (Henneberger et al., 2010; Henneberger and Rusakov, 2012). Briefly, whole-cell recordings in astrocytes were obtained using standard patch pipettes (3-4 MΩ) filled with an intracellular solution containing (in mM) KCH_3_O_3_S 135, HEPES 10, Na_2_-Phosphocreatine or di-Tris-Phosphocreatine 10, MgCl_2_ 4, Na_2_-ATP 4, Na-GTP 0.4 (pH adjusted to 7.2 using KOH, osmolarity 290-295). Cell-impermeable dyes Fluo-4 (200 μM, Invitrogen) and AF 594 hydrazide (20-100 μM) were routinely added to the intracellular solution, unless indicated otherwise. Where specified, bumetanide (20 μM) or S3 peptide fragment (200 μM, Anaspec) was added to the intracellular solution. Passive astrocytes were identified by their small soma size (∼10 μm; visualized in the AF emission channel), low resting potential (below −80 mV without correction for the liquid-junction potential), low input resistance (< 10 MΩ), passive (ohmic) properties and characteristic morphology of the arbour (Figures 1B, S1A, and S3B). Astrocytes were either held in voltage clamp mode at their resting membrane potential or in current clamp. Where specified, the intracellular free Ca^2+^ concentration was clamped to a steady-state level of 50-80 nM by adding 0.45 mM EGTA and 0.14 mM CaCl_2_ to the intracellular solution (calculation by WebMaxChelator, Stanford).

#### LTP induction *ex vivo*

Where indicated, an extracellular recording pipette was placed immediately adjacent to the astrocyte under investigation visualized in the AF channel (Figure 1B). Synaptic responses were evoked by orthodromic stimulation (100 μs, 20-100 μA) of Schaffer collaterals using either a bipolar or coaxial stimulation electrode placed in the *stratum radiatum* > 200 μm away from the recording electrodes. Field EPSPs (fEPSPs) were recorded using a standard patch pipette filled with the extracellular solution. In some experiments, astrocytic EPSCs (a-fEPSCs) or field EPSPs (a-fEPSPs) were also recorded using the cell patch pipette (Henneberger and Rusakov, 2012): the astrocytic readout was fully consistent with extracellular fEPSPs (Figure S3C). The baseline stimulus intensity was set at ∼50% of the maximal response, stimuli were applied every 30 s for at least 10 minutes before LTP was induced using three trains of high-frequency stimulation (HFS, 100 pulses at 100 Hz) 60 s apart. The slope of fEPSPs was monitored afterward for at least 30 minutes. See sections below for LTP induction protocols used in specific experiments, such as through glutamate uncaging or using a ‘chemical cocktail’.

#### 2PE imaging of astroglia *ex vivo*

Over the course of this study we used a Radiance 2100 (Zeiss-Biorad), FV1000MP (Olympus), Femto3D-RC (Femtonics, Budapest), or Femto2D (Femtonics, Budapest) microscope and a Scientifica imaging system optically linked to femtosecond pulse lasers MaiTai (SpectraPhysics-Newport) or Vision S (Coherent) and integrated with patch-clamp electrophysiology, as detailed earlier (Henneberger et al., 2010; Jensen et al., 2019; King et al., 2020; Zheng et al., 2015). Once in whole-cell mode, dyes normally equilibrated across the astrocyte tree within 5-10 min. In astrocyte morphology time-lapse experiments, astrocytes loaded with fluorescence indicators were routinely imaged in frame mode at a nominal resolution of ∼0.1 μm / pixel (512x512 pixels, 25x Olympus objective /NA1.05) in the red emission channel (540LP / 700SP filter; λ_x_^2P^ = 800 nm). To minimize photodamage, only a single focal section through the soma (average of three) was acquired at a laser intensity of 3-6 mW under the objective with careful adjustment of the z-position. Patch-clamp experiments were controlled and analyzed using pCamp10 (Molecular Devices, RRID SCR_011323) and WinWCP Strathclyde Electrophysiology Software (RRID SCR_014270). Image acquisition used MES.4/5 (Femtonics Ltd., RRID SCR_018309), image analyses involved ImageJ (NIH, RRID SCR_003070), *ad hoc* routines written in MATLAB (Mathworks, RRID SCR_001622) and OriginPro (OriginLab, RRID SCR_014212).

#### iGluSnFR transduction in astroglia

To express the optical glutamate sensor iGluSnFR (Marvin et al., 2013) in astrocytes, an AAV virus expressing iGluSnFR under a GFAP promoter (AAV1.GFAP.iGluSnFr.WPRE.SV40; Penn Vector Core, PA, USA), we used bilateral injection into the ventral hippocampus. C57BL6/N mice (4 weeks old, Charles Rivers Laboratories) were injected intra-peritoneally with a ketamin/medotomidine anesthesia (100 and 0.25 mg per kg body weight in NaCl, injection volume 0.1 mL per 10 g body weight, ketamin 10%, betapharm; Cepotir 1 mg/ml, CPPharma). First, the head fur was removed and the underlying skin disinfected. After ensuring that the animal was under deep anesthesia, the head was fixed in a stereotactic frame (Model 901, David Kopf Instruments). After making an incision, bregma was localized. Next, the coordinates for the ventral hippocampus (relative to bregma: anterior −3.5 mm, lateral -/+3 mm, ventral −2.5 mm) were determined and the skull was locally opened with a dental drill. Under control of a micro injection pump (100 nl/min, WPI) 1 μl viral particles were injected with a beveled needle nanosyringe (nanofil 34G BVLD, WPI). After retraction of the syringe, the incision was sutured using absorbable thread (Ethicon). Finally, the anesthesia was stopped by i.p. application of atipamezol (2.5 mg per kg body weight in NaCl, injection volume 0.1 mL per 10 g body weight, antisedan 5 mg/ml, Ventoquinol). To ensure analgesia, carprofen (5 mg/kg in NaCl, injection volume 0.1 ml/20 g body weight, Rimadyl 50 mg/ml, Zoetis) was injected subcutaneously directly, 24h and 48h after the surgery.

#### iGluSnFR transduction in neurons

C57BL/6 mice (3 - 4 weeks of age), male and female, were prepared for aseptic surgery and anaesthetised using isoflurane (5% v/v induction, 1.5 - 2.5% maintenance). The scalp was shaved and disinfected using three washes of topical chlorhexidine. The animal was secured in a stereotaxic frame (David Kopf Instruments, CA, USA) and loss of pedal reflexes was confirmed prior to surgery. Body temperature was maintained at 37.0 ± 0.5°C using a feedback rectal thermometer and heating blanket. Perioperative analgesics were administered (subcutaneous buprenorphine, 60 μg kg^-1^, topical lidocaine/prilocaine emulsion, 2.5%/2.5%) before ocular ointment (Lacri-lube, Allergan, UK) was applied to the eyes. A small midline incision was made and superficial tissue resected to expose the skull. A craniotomy of approximately 1 mm diameter was performed over the right hemisphere using a high-speed hand drill (Proxxon, Föhren, Germany), at a site overlying the medial hippocampus. Stereotactic coordinates were 60% of the anteroposterior distance from bregma to lambda and 2.5 mm lateral to midline. Upon exposure, a warmed, sterile saline solution was applied to exposed cortical surface during the procedure.

Pressure injections of AAV9 hSyn iGluSnFR (totalling 0.1 - 1 × 10^10^ genomic copies in a volume not exceeding 200 nL, supplied by Penn Vector Core, PA, USA) were carried out using a pulled glass micropipette stereotactically guided to a depth of 1.3 mm beneath the cortical surface, at a rate of approximately 1 nL sec^-1^. The total injection volume was delivered in three steps, reducing depth by 100 μm at each step. Once delivery was completed, pipettes were left in place for 5 minutes before being retracted. The surgical wound was closed with absorbable 7-0 sutures (Ethicon Endo-Surgery GmbH, Norderstedt, Germany) and the animal was left to recover in a heated chamber. Meloxicam (subcutaneous, 1 mg kg^-1^) was subsequently administered once daily for up to two days following surgery. Mice were killed by transcardial perfusion with ice-cold sucrose-enriched slicing medium (in mM, 105 sucrose, 60 NaCl, 2.5 KCl, 1.25 NaH_2_PO_4_, 26 NaHCO_3_, 15 glucose, 1.3 ascorbic acid, 3 Na pyruvate, 0.5 CaCl_2_ and 7 MgCl_2_, saturated with 95% O_2_ and 5% CO_2_) after a 2 - 4 week AAV incubation period and acute hippocampal slices prepared for imaging and electrophysiological recordings as below.

#### Viral transduction of astroglial GFP

An AAV virus expressing the astroglial GFP (AAV5.GfaABC1D.Pi.lck-GFP.SV40, supplied by Penn Vector Core, PA, USA) was injected into the cerebral ventricles of neonates. C57BL/6J mice (P0-P1), male and female, were prepared for aseptic surgery and maintained all time while being away from the mothers in a warm environment to eliminate risk of hypothermia in neonates. Intracerebroventricular (ICV) injections were carried out after a sufficient visualization of the targeted area to ensure a proper injection. Viral particles (totalling 5 × 10^9^ genomic copies in a volume 2 μl) were injected using a glass Hamilton microsyringe, 2 μl/hemisphere, at a rate not exceeding of 0.2 μl/s, 2 mm deep, perpendicular to the skull surface, guided to a location approximately 0.25 mm lateral to the sagittal suture and 0.50–0.75 mm rostral to the neonatal coronary suture. Once delivery was completed, microsyringe was left in place for 20-30 s before being retracted. Pups received ICV injections were kept as a group of litters and returned to the mother in their home cage.

#### Dual transduction in the barrel cortex

C57BL/6 mice (3 - 4 weeks of age), male and female, were prepared as above for neuronal expression of iGluSnFR. During the procedure, two craniotomies of approximately 1 mm diameter were performed over the right hemisphere using a high-speed hand drill (Proxxon, Föhren, Germany), at sites overlying the ventral posteromedial nucleus of the thalamus (VPM) and the barrel cortex (S1BF). The entire microinjection into the VPM was completed prior to performing the second craniotomy over S1BF. Stereotactic coordinates for VPM injections were −1.8 mm and 1.5 mm along the anteroposterior and mediolateral axes, respectively. Two injection boluses was delivered at 3.0 and 3.2 mm beneath the dural surface. For S1BF injections, the coordinates were −0.5 mm and 3.0 mm along the anteroposterior and mediolateral axes, respectively, delivering a single bolus at a depth of 0.6 mm. A warmed saline solution was applied to exposed cortical surface during the procedure.

Pressure injections of AAV9 hSyn.GCaMP6f (totalling 1 × 10^10^ genomic copies in a volume not exceeding 200 nL, supplied by Penn Vector Core, PA, USA) and AAV5 GfaABC1D tdTomato (0.5 × 10^10^ genomic copies, in a volume not exceeding 200 nL, supplied by Penn Vector Core, PA, USA) were carried out using a glass micropipette at a rate of 1 nL sec^-1^, stereotactically guided to the VPM and S1BF, respectively, as outlined above. Once delivery was completed, pipettes were left in place for 5 minutes before being retracted. The surgical wound was closed and the animal recovered as outlined above for neuronal expression of iGluSnFr. Meloxicam (subcutaneous, 1 mg kg^-1^) was administered once daily for up to two days following surgery. Mice were subsequently prepared for cranial window implantation approximately 2 weeks later.

#### Cranial window implantation

Mice were prepared for aseptic surgery and secured in a stereotaxic frame as before during the viral transduction procedure. Once secured and under stable anesthesia (isoflurane, maintenance at 1.5 - 2%), a large portion of the scalp was removed to expose the right frontal and parietal bones of the skull, as well as the medial aspects of the left frontal and parietal bones. The right temporalis muscles were reflected laterally to expose the squamous suture, to facilitate cement bonding during fixation of the cranial window implant. The exposed skull was coated with Vetbond (3M, MN, USA) and a custom-made headplate was affixed over the S1BF. The assembly was then secured with dental cement (SuperBond, Sun Medical Co. Ltd., Japan). Once the bonding agents had cured, the animal was removed from the stereotaxic frame and it’s headplate was secured in a custom-built head fixation frame. A craniotomy of approximately 4 mm diameter was carried out over the right somatosensory cortex, centered over the S1BF injection site. Immediately prior to removal of the skull flap, the surface was superfused with warmed aCSF (in mM; 125 NaCl, 2.5 KCl, 26 NaHCO_3_, 1.25 Na_2_HPO_4_,18 Glucose, 2 CaCl_2_, 2 MgSO_4_; saturated with 95% O_2_ / 5% CO_2_, pH 7.4). The dura was resected using a combination of 26G needles (tapped against a hard surface to introduce a curved profile), fine-tipped forceps (11252-40, Fine Science Tools, Germany) and 2.5 mm spring scissors (15000-08, Fine Science Tools, Germany), taking care not to penetrate to the pia mater. Once the dura was removed, a previously-prepared coverslip consisting of a 3 mm diameter round coverglass affixed beneath a 4 mm diameter round coverglass (Harvard Apparatus UK, affixed using a UV-curable optical adhesive (NOA61), ThorLabs Inc., NJ, USA) was placed over the exposed cortex. Slight downward pressure was applied to the coverslip using a stereotactically guided wooden spatula that was previously severed and sanded to allow some flexibility and preclude excessive force. The superfusion was discontinued and excess aCSF was removed using a sterile surgical sponge, taking care not to wick fluid from beneath the cranial window. The coverslip was then secured with VetBond and dental cement, sequentially. Once cured, the animal was recovered in a heated chamber and returned to its homecage when ambulatory. Post-operative care was administered as before during the viral transduction procedure.

#### Multiplexed 2PE imaging *in vivo*

Two-photon excitation was carried out using a wavelength multiplexing suite consisting of a Newport-Spectraphysics Ti:sapphire MaiTai tunable IR laser pulsing at 80 MHz and a Newport-Spectraphysics HighQ-2 fixed-wavelength IR laser pulsing at 63 MHz, as detailed earlier (Mishra et al., 2016; Reynolds et al., 2019; Zheng et al., 2015). The laser lightpaths were aligned (though not synchronized) before being point-scanned using an Olympus FV1000 with XLPlan N 25x water immersion objective (NA 1.05). During imaging, animals were lightly anaesthetised (fentanyl, 0.03 mg kg^-1^, midazolam, 3 mg kg^-1^, and medetomidine, 0.3 mg kg^-1^) and secured under the objective on a custom-built stage via the previously affixed headplate.

Initial acquisitions were performed with both lasers illuminating the tissue at 910 nm and 1040 nm, respectively, in order to locate active thalamocortical boutons in S1BF within the arbor of tdTomato-positive cortical astrocytes. Brief 5 s, 3 Hz pulses of nitrogen were directed at the contralateral whiskers to determine responsive regions of interest. Measurements were performed in L1 and L2/3, at depths of 50 - 150 nm. For bouton recordings, framescans of 4 - 20 Hz were performed, with a pixel dwell time of 2 μs and a mean laser power of 30 mW at the focal plane. Upon identification of suitable astrocytes, we sampled the baseline VF. Except when needed for illustrative purposes, illumination by the tunable IR laser (910 nm, to excite GCaMP6f) was occluded at this stage, in order to limit photobleaching. High-resolution z stacks, incorporating 1 or more astrocytes, were taken every 2.5 minutes, for 15 - 20 minutes. Z stacks were 512 × 512 pixels, with a pixel size of 0.25 - 0.5 μm and an interval size of 1.5 - 2.5 μm. Sensory-evoked synaptic potentiation within the barrel cortex was then induced as previously described (Gambino et al., 2014), via a contralateral rhythmic whisker stimulation (RWS, 120 s, 3 Hz). Sampling of z stacks, covering the same cortical area, was continued for 30 - 45 minutes following the RWS. The same regions were sampled again one week later, before and after an ipsilateral RWS, to serve as control VF measurements. To determine VF *in vivo*, stacks were coded (to blind experimenters) and motion-corrected using MATLAB. Fluorescence values for the astrocytic soma and 2 - 4 ROIs within its arbor, from the same focal plane, were tabulated. Sampling of fluorescence from the primary astrocytic branches was avoided as pilot data indicated that VF changes within such branches was negligible. Values for each ROI were averaged to give cell-specific ratiometric fluorescence values, which were normalized to yield relative changes in VF.

#### Monitoring PAP VF

Astrocyte tissue volume fraction (VF) was monitored to detect structural changes of fine astrocyte branches smaller than the diffraction limit (200-300 nm for diffraction-limited 2PE imaging). VF was obtained by normalizing the background-corrected fluorescence of AF 594, AF 488, intracellularly expressed EGFP, or tdTomato, as indicated, to somatic values, where 100% of the tissue is occupied by the astrocyte (Figures 1A, 1B, and S1A–S1C). The VF values obtained with this approach were not affected by dye escape through gap-junctions or hemichannels (Figure S1C).

#### Fluorescence recovery after photobleaching (FRAP) experiments

FRAP of AF 594 was used to quantify changes of intracellular diffusivity in astrocytes. Fluorescence recordings were obtained in line-scan mode (500 Hz, line placed quasi-randomly through the astrocyte arbour) at an increased laser power of 15-20 mW under the objective to induce substantial bleaching of AF 594, as further detailed in (Anders et al., 2014).

#### Optical measurements of extracellular diffusivity

The effective diffusivity of fluorescent dyes was determined using a point-source diffusion method as previously described (Savtchenko and Rusakov, 2005; Zheng et al., 2008). Briefly, a bolus of fluorescent dye (AF 594 hydrazide, 50 μM in extracellular solution) was ejected from a patch pipette into the CA1 *stratum radiatum* neuropil by a pressure pulse (0.8 bar, 2-6 ms). The diffusion spread of the dye was traced by scanning along a line in front of the ejection pipette (∼300-1000 Hz; Figure S1F). Fluorescence life profiles for each time point were fitted with the Gaussian function $\exp\left({\left(x-x_{c}\right)}^{2}/4w\right)$, with *w* = *D*_*eff*_ *t*, where *x* is the pixel co-ordinate within the linescan, *x*_*c*_ the puff source (pipette tip) co-ordinate, *D*_*eff*_ the effective diffusivity and *t* is time since the puff. *D*_*eff*_ is then obtained by linear fitting of *w*(*t*) (Figure S1G). All analyses were performed using MATLAB (Mathworks). Measurements were repeated every 10 minutes. Field EPSPs were evoked by Schaffer collateral stimulation (see above) and recorded through another field pipette < 150 μm away from the puff pipette. In a subset of recordings, LTP was induced after 10 minutes of baseline recording.

#### STED microscopy in organotypic slices

Organotypic hippocampal slice cultures were prepared from 5-7 day pups of Thy1-YFP transgenic mice, in accordance with the French National Code of Ethics on Animal Experimentation and approved by the Committee of Ethics of Bordeaux (No. 50120199). As described before (Nägerl et al., 2004), cultures were prepared using the roller tube method (Gähwiler method). First, pups were decapitated. Next, brains were removed, hippocampus dissected (in cooled Gey’s Balanced Salt Solution, GBSS) and 350 μm coronal slices were sectioned using a tissue chopper (McIlwain). After 30-60 minutes rest at 4°C in GBSS, each half slice was mounted on a glass coverslip coated with heparinized chicken plasma (10 μl, Sigma). Thrombin (Merck) was added to coagulate the plasma and to allow the slice to adhere to the coverslip. After 30 minutes at room temperature, each coverslip was inserted into a delta tube (Nunc) before adding 750 μl culture medium containing: 50% Basal Medium Eagle (BME, GIBCO), 25% Hanks’ Balanced Salt solution (HBSS, GIBCO), 25% of heat inactivated horse serum (GIBCO) supplemented with glutamine to a final concentration of 1mM and glucose to a final concentration of 11 g/l (Sigma). Finally, slices were cultivated during 5-6 weeks in tubes placed on a roller-drum incubator set at 35°C in dry air with a rotation rate of ∼10 revolutions per hour. The experimental day, the slice was transferred to a submersion-type recording chamber perfused (2 ml/min) with ACSF at 31°C saturated with 95%O_2_/5%CO_2_ and containing (in mM): NaCl 119, KCl 2.5, NaH2PO4 1.25, NaHCO3 26, Trolox 1.5 and 10 glucose (pH 7.4; osmolarity 295-298) in the presence of 1.3 mM Mg^2+^ and 2 mM Ca^2+^.

To enable STED microscopy studies, as described previously (Tønnesen et al., 2011), a home-built STED microscope was constructed around the base of an inverted confocal microscope (DMI 6000 CS Trino, Leica, Mannheim, Germany) using a glycerin objective with a high numerical aperture and equipped with a correction color (PL APO, CORR CS, 63x, NA 1,3; Leica), providing an optical resolution of at least 70 nm in x-y tens up to 50 μm below the tissue surface. A pulsed-laser diode (PDL 800-D, Picoquant, Berlin, Germany) was used to deliver excitation pulses at 485 nm wavelength with 90 ps duration. Furthermore, an optical parametric oscillator (OPO BASIC Ring fs RTP, APE, Berlin, Germany) pumped by a Ti:Sapphire laser (MaiTai, Spectra-Physics, Darmstadt, Germany), operating at 80 MHz produced a pulsed STED beam centered at a wavelength of 592 nm, to quench the fluorescence. The maximal power of the STED beam going into the back aperture of the objective was 12 mW. Both, excitation and STED pulses were synchronized at 80 MHz by externally triggering the laser diode and optimizing the relative delay using an electronic delay generator. The fluorescence signal was first separated from the excitation light by a dichroic mirror (499-nm long-pass), then cleaned with a 525/50 band-pass filter, spectrally separated by a dichroic mirror (514-nm long-pass), and finally imaged onto two multimode optical fibers connected to avalanche photodiodes (SPCM-AQR-13-FC, PerkinElmer, Waltham, MA).

Image acquisition was controlled by the software IMSpector (Abberior Instruments Development Team, Imspector Image Acquisition & Analysis Software v0.1, http://imspector.abberior-instruments.com). The pixel dwell time was 15 μs with a pixel size of 19.53 nm. Typically, 2 μm stacks, with nine z sections, 220 nm apart were acquired. As described before (Tønnesen et al., 2011), YFP (in neurons) and AF 488 (in astrocytes) were spectrally detected using a 514 nm long-pass emission filter. Effective color separation was achieved offline by linear un-mixing of the fluorescence channels (using a plugin from ImageJ) after deconvolution (3 iterations) using Huygens Professional (SVI). All morphometric analyses were done on deconvolved image sections of the two unmixed color channels. To determine spine head width, a 3-pixel thick line was manually positioned through the largest part of the spine head, and the full width at half maximum (FWHM) as a measure of spine size was extracted from the line profile. Astrocytic processes and spines were considered to be in close proximity if the visible distance between their edges (as determined by the FWHM of a line profile laid across the point of shortest distance) was equal or less than 20 nm, corresponding to one pixel. Conversely, for separations larger than 1 pixel, the astrocytic process and spine were not considered to be in close proximity.

#### Fast fixation and DAB staining

In a subset of experiments, we loaded a recorded astrocyte with biocytin, and after the experiment the slices were rapidly fixed (by submersion) with 1.25% glutaraldehyde and 2.5% paraformaldehyde in 0.1 M PB (phosphate buffer, pH 7.4), to be kept overnight, infiltrated in 10% sucrose in PB for 10 min and then in 20% sucrose in PB for 30 min. Infiltrated slices were consequentially freeze-thaw in liquid freon and liquid nitrogen for 3 s each to gently crack intracellular membranes and embedded in 1% low gelling temperature agarose in PB (Sigma-Aldrich, USA). Embedded slices were sectioned at 50 μm on a vibrating microtome (VT1000; Leica, Milton Keynes, UK). 50 μm sections were incubated in 1% H2O2 in PB for 20 min to eliminate blood background, washed with 0.1 M TBS (tris buffer saline, pH 7.4) and incubated with ABC solution (VECTASTAIN ABC, Vector laboratories, USA) for 30 min at room temperature. Next section were washed with 0.1M TB (tris buffer, pH 7.4), pre-incubated with DAB (3,3′-Diaminobenzidine tablets - Sigma-Aldrich, USA) solution (10 mg DAB tablet + 40 mL TB) for 30 min at room temperature in dark and finally incubated with DAB+ H_2_O_2_ solution (5 μL of 33% H_2_O_2_ + 25 mL of DAB solution) for 10-20 min at room temperature in dark. The DAB stained sections were washed in PB, post-fixed in 2% osmium tetroxide and further processing and embedding protocols were essentially similar to those reported previously (Medvedev et al., 2010). Briefly, the tissue was dehydrated in graded aqueous solutions of ethanol (30%–100%) followed by 3 times in 100% acetone, infiltrated with a mixture of 50% epoxy resin (Epon 812 ∕ Araldite M) and 50% acetone for 30 min at room temperature, infiltrated in pure epoxy resin, and polymerized overnight at 80°C. Sections in blocks were coded and all further analyses were carried out blind as to the experimental status of the tissue.

#### 3D electron microscopy

Serial sections (60–70 nm thick) were cut with a Diatome diamond knife as detailed and illustrated earlier (Medvedev et al., 2010; Popov et al., 2004, 2005), and systematically collected using Pioloform-coated slot copper grids (each series consisted of up to 100 serial sections). Sections were counterstained with 4% uranyl acetate, followed by lead citrate. Finally, sections were imaged in *stratum radiatum* area of CA1 (hippocampus) using an AMT XR60 12 megapixel camera in a JEOL 1400 electron microscope. Serial sections were aligned using SEM. Align 1.26b (provided by John Fiala, Boston University). 3D reconstructions of DAB stained astrocyte processes and the adjacent dendritic spines were performed in Trace 1.6b software (https://synapses.clm.utexas.edu/). Dendritic spines were categorized according to (Harris et al., 1992; Peters and Kaiserman-Abramof, 1970); since 90%–95% of excitatory synapses in CA1 area of hippocampus are located on either thin or mushroom dendritic spines only the mushroom (n = 88) and thin (n = 243) spines were reconstructed and analyzed. 3D reconstructions of segmented astrocytic processes and dendritic spines were imported to 3D-Studio-Max 8 software for rendering of the reconstructed structures.

#### Astroglial coverage in 3D EM

To analyze astroglial coverage of synapses, a set of virtual 100 nm thick concentric spherical shells (Figure 3D) was arranged *in silico* around each reconstructed PSD using custom-made software. The volume of each shell as well as the volume and surface area of astrocytic segments inside each shell were computed to estimate the volume fraction (VF) occupied by astrocyte processes (astrocyte volume / total shell volume) and the surface area of astrocyte, throughout concentric shell between centered at 0-0.5 μm around the centroid of each individual PSD. In some cases, we also carried out additional analyses using curvilinear 3D shells reproducing the contours of each PSD; the results were qualitatively identical. All data from digital reconstructive analyses were evaluated to obtain one value for each individual slice taken from individual animals (there were n = 3 preparations in each group), in each dataset. ANOVA tests were used to examine differences between specific animal groups (implemented through Origin Pro 7.5). Data were presented as mean ± SEM (n = 3 animals per group).

#### Chemical LTP induction

The classical ‘chemical’ LTP (cLTP) was induced by perfusing the acute slice for 10-15 min with the Mg-free ACSF solution containing 4 mM CaCl_2_ (Sigma), 0.1 μM rolipram (Cayman Chemical Company), 50 μM forskolin (Cayman Chemical Company) and 50 μM picrotoxin (Sigma) (Otmakhov et al., 2004). This treatment increases the level of cAMP and that of network activity leading to a tetanic-like stimulation in bulk that potentiates the majority of excitatory synapses.

#### Three-color 3D dSTORM

We used a modified protocol described by us previously (Heller et al., 2017, 2020). Following induction of cLTP, acute hippocampal slices were fixed with 4% PFA in PBS for 20 min and washed with PBS three times for 20 min each before being embedded in 4% agarose. Slices were then resliced into 30 μm sections and kept free-floating in PBS; non-reacted aldehydes were quenched in 0.1% NaBH_4_ in PBS for 15 min; washed thrice for 5 min with PBS; autofluorescence was quenched with 10 mM CuSO_4_ in 50 mM NH_4_Cl, final pH = 5 for 10 min; washed with H_2_O thrice quickly and once with PBS (5 min). Permeabilisation and blocking was carried out with PBS-S (0.2% saponin in PBS) supplemented with 3% BSA for at least 3 hours; incubated with primary antibody (see below) in PBS-S overnight at 4°C; washed trice with PBS-S; incubated with secondary antibody (see below) in PBS-S for two hours; washed with PBS-S twice for 10 min and with PBS twice for 10 min; post-fixed with 4% PFA in PBS for 30 min; washed with PBS thrice for 10 min; incubated in Scale U2 buffer (Hama et al., 2011) (4 M urea, 30% Glycerol and 0.1% Triton X-100 in water) at 4°C until being prepared for imaging.

Primary antibodies were for: presynaptic protein Bassoon (Mouse, SAP7F407, Recombinant rat Bassoon, Novus, NB120-13249, RRID AB_788125, dilution 1:500), postsynaptic protein Homer1 (Rabbit, polyclonal, Recombinant protein of human homer (aa1-186), Synaptic Systems, 160003, RRID AB_887730, dilution 1:500), glial glutamate transporter GLT1 (Guinea pig, Polyclonal, Synthetic peptide from the C terminus of rat GLT1, Merck, AB1783, RRID AB_90949, dilution 1:500). Secondary antibodies were: anti-mouse IgG (Donkey, CF568-conjugated, Biotium, 20105, RRID AB_10557030, dilution 1:500), anti-rabbit IgG (Goat, Atto488-conjugated, Rockland, 611-152-122S, RRID AB_10893832, dilution: 1:500), anti-guinea pig IgG (Donkey, Alexa647-conjugated, Jackson ImmunoResearch Labs, 706-606-148, RRID AB_2340477, dilution: 1:500).

To obtain spatial patterns of individual proteins in the synaptic microenvironment, we employed the single-molecule localization microcopy (SMLM) technique direct stochastic optical reconstruction microscopy (dSTORM) (Endesfelder and Heilemann, 2015) . Images were recorded with a Vutara 350 microscope (Bruker). The targets were imaged using 640 nm (for Alexa647), 561 nm (for CF568) or 488 nm (for Atto488) excitation lasers and a 405 nm activation laser. We used a photoswitching buffer containing 100 mM cysteamine and oxygen scavengers (glucose oxidase and catalase) (Metcalf et al., 2013). Images were recorded using a 60x-magnification, 1.2-NA water immersion objective (Olympus) and a Flash 4.0 sCMOS camera (Hamatasu) with frame rate at 50 Hz. Total number of frames acquired per channel ranged from 5000-20000. Data were analyzed using the Vutara SRX software (version 6.02.05) and a custom-written script for MATLAB. Single molecules were identified by their continued emission frame-by-frame after removing the background. Identified particles were then localized in three dimensions by fitting the raw data with a 3D model function, which was obtained from recorded bead datasets. The experimentally achievable image resolution is 20 nm in the *x-y* plane and 50 nm in the *z* direction; in tissue sections we routinely achieved *x-y* resolution of 58.0 ± 7.1 and *z-*resolution of 73 ± 5.8 nm.

#### LTP induction by 2PE glutamate spot-uncaging

We used a combined two-photon uncaging and imaging microscope (Olympus, FV-1000MPE) powered by two Ti:Sapphire pulsed lasers (Chameleon, Coherent, tuned to 720 nm for uncaging and MaiTai, Spectra Physics, tuned to 840 nm for imaging). The intensity of the imaging and uncaging laser beams under the objective was set to 5 mW and 12-17 mW, respectively. CA1 pyramidal neurons and astrocytes were loaded with Fluo-4 (200 μM) and AF 594 (100 μM) and held in current-clamp mode. The MNI-glutamate was applied in the bath at 2.5 mM. The stimulation protocol was delivered > 30 μm from the cell soma and included three series of 100 × 1ms pulses at 100Hz, 60 s apart. The uncaging spot was placed ∼1μm from the identifiable small process in astrocytes or the dendritic spine head in patched and visualized CA1 pyramidal neurons.

To test whether this protocol elicited LTP, CA1 pyramidal neurons were recorded in whole-cell patch clamp, as described in the text, and EPSCs were elicited by 1 ms uncaging pulses delivered every 3 min. After a 10 min baseline, the neuron was held in current clamp (−60 to −65 mV, as in freely-moving rats) (Epsztein et al., 2010) and LTP was induced using the glutamate uncaging protocol. Once the induction protocol had been completed, EPSCs were monitored in voltage clamp for another 30 min.

For IP_3_ uncaging, 400 μM NPE-caged IP_3_ (D-Myo-Inositol 1,4,5-Triphosphate, P4(5)-(1-(2-Nitrophenyl)ethyl) ester, Life Technologies) were added to the internal solution. The uncaging protocol consisted of 3-5 cycles (200 ms apart) of 5-10 ms pulses on 4-5 points, repeated 3 times every 60 s. To test the effect of glutamate and IP_3_ uncaging on astrocyte morphology, astrocytes located in the *stratum radiatum* of CA1 were loaded with Fluo-4 (200 μM) and AF 594 (100 μM).

In baseline conditions, and 30-40 min after the glutamate-uncaging LTP induction protocol, Z stacks of the same region of the astrocyte were collected every 60-120 s. The intracellular Ca^2+^ response to glutamate and IP_3_ uncaging was recorded using frame-scans in astrocytes (Figures 4A and 5E) and linescan recordings in dendritic spines of CA1 pyramidal cells and expressed as Δ*G/R* values (green/red ratio; Fluo-4 fluorescence normalized to the AF 594 signal, Figures S5A and S5B).

#### Probing ephrins and extracellular matrix

The candidate morphogenic signals that could be invoked during LTP induction involve signaling molecules of the extracellular matrix (ECM) (Dityatev and Rusakov, 2011) or the ephrin/Eph-dependent neuron-astrocyte signaling attributed to astrocyte-dependent stabilization of newly formed dendritic protrusions (Nishida and Okabe, 2007). The protocol for catalytic removal of chondroitin sulfate (and side chains of proteoglycans) with Chondroitinase ABC (0.5U/ml, 45 min, 33°C) has been established and validated by us previously (Kochlamazashvili et al., 2010). Similarly, the blockade of EphA4 activity with EphA4-Fc (10 μg/ml) using previously tested protocols was carried out in accord with the reported procedures (Murai et al., 2003). Because degrading the ECM’s hyaluronic acid with hyaluronidases interfered with LTP induction (Kochlamazashvili et al., 2010) such experiments were not included in the present study. Suppressing NKCC1 activity in the recorded astrocyte was performed through intracellular dialysis of bumetanide (20 μM) (Migliati et al., 2009).

#### Glutamate imaging with FLIPE600n

We modified FLIPE600n (Okumoto et al., 2005) to contain a biotin tag for immobilization of the sensor in the tissue, as described previously (Whitfield et al., 2015; Zhang et al., 2018). A nucleotide sequence coding for the biotin tag was synthetized *de novo* (Epoch Life Science), amplified using PCR and then inserted into pRSET FLIPE-600n (Addgene #13537, courtesy of Wolf B. Frommer) using BamHI restriction site.

bFLIPE600n reports glutamate levels through a FRET mechanism, by changing the fluorescence intensity ratio *R* = ECFP/Venus. Calibration of the bFLIPE600n sensor using 2PE was first done in free solution (Figures S5E and S5F). bFLIPE600n in PBS (3-4 μM, pH 7.4) was placed in a meniscus under the microscope objective. Increasing amounts of glutamate (dissolved in PBS, pH adjusted to 7.4) were added and changes in the ECFP/Venus emission ratio were calculated offline. For experiments in acute slices, 30-40 μM bFLIPE600n were preincubated with 5-7 μM streptavidin in PBS at 4°C for at least 12 h. A standard patch pipette (2-4 MΩ resistance) was then backfilled with the sensor solution and bFLIPE600n was gently injected into the CA1 s. *radiatum* of biotinylated slices (see above and (Whitfield et al., 2015; Zhang et al., 2018)) at 70-100 μm depth applying light positive pressure for 10-20 s. Sensor levels were allowed to equilibrate for 15 min before recordings started at a depth of 50-60 μm below the slice surface. Schaffer collateral stimulation was done as described above except that the stimulation intensity was ∼50% of the one inducing near-maximal fEPSP responses.

#### Glutamate imaging with iGluSnFR

iGluSnFR was expressed in the CA1 region of the hippocampus as described above. A iGluSnFR-expressing CA1 pyramidal neuron was loaded with 100 μM AF 594 to visualize dendritic spines. The iGluSnFR fluorescence was monitored in linescan mode (λ_x_^2P^ = 910 nm, 500 Hz) following MNI-glutamate uncaging (1 ms pulse, 2.5 mM in the bath). The linescan was positioned near the closest dendritic spines head, parallel to the dendritic stem (Figure 6A). In baseline conditions, three linescan images were recorded 3 min apart and averaged. LTP was induced with 2PE uncaging of glutamate as described above. 5-10 min following LTP induction, five linescan images were recorded every five minutes for averaging.

In each linescan image, two ∼30 ms long ROI bands were selected for analyses, one shortly before the spot-uncaging onset (background iGluSnFR fluorescence profile *F*_*0*_ along the linescan axis *x*, *F*_*0*_(*x,t*)) the and one ∼10 ms after (Figure 6B). The pixel brightness values (originally recorded gray scale) in these iGluSnFR linescan images were (i) averaged along the timeline *t*, and (ii) among the pre-uncaging and the post-uncaging groups in each trial, thus giving average profiles *F^∗^*_*0*_(*x*) and *F^∗^*(*x*), respectively, for trials before and after LTP induction. Thus, in each trial the iGluSnFR glutamate signal profile was obtained as a pixel-by-pixel image (vector) operation (*F^∗^*(*x*)*-F^∗^*_*0*_(*x*)) */ F^∗^*_*0*_(*x*) giving the glutamate-bound iGluSnFR brightness distribution *ΔF/F*_*0*_ (*x*) along the linescan axis near the uncaging spot. The distribution *ΔF/F*_*0*_ (*x*) along x axis (distance) was best-fit approximated with a Gaussian centered at the uncaging spot, with the Gaussian amplitude and variance being free parameters (OriginPro, Origin Lab Corp, MA).

#### Two-pathway cross-talk experiments

The NMDAR-mediated synaptic cross-talk was probed by taking advantage of the use-dependency of the NMDAR inhibitor MK801, as described in detail earlier (Scimemi et al., 2004). CA1 pyramidal cells where held in voltage clamp to record EPSCs in response to stimulation of two independent synaptic CA3-CA1 pathways (see Figure 4C for an illustration, GABA receptors blocked as described above). While individual pathways displayed a robust (same-pathway) paired-pulse facilitation of 75.4 ± 6.1% (n = 54, p < 0.001; inter-stimulus interval 50 ms), the facilitation was approximately five times lower between the pathways (16.5 ± 2.9%, p < 0.0001) thus indicating that these pathways do not interact presynaptically by more than ∼20%. Separation of pathways was helped by making an additional cut into the *stratum radiatum* in parallel to the pyramidal cell layer. AMPAR-mediated EPSCs were recorded at a holding potential of −70 mV for 10-15 minutes. In a subset of experiments LTP was induced on one or both pathways (HFS, see above). NMDAR-mediated EPSCs of the same pathways were then recorded by clamping the cell to −20 mV and inhibiting AMPAR with 10 μM NBQX. MK801 (4 μM) was bath-applied after another baseline period. Stimulation of the test pathway was then stopped and resumed after 20 minutes. EPSCs were evoked at 0.1 Hz throughout the experiment. Synaptic cross-talk was quantified at the test pathway by calculating the reduction of NMDAR-mediated EPSC amplitudes in the absence of test pathway stimulation relative to baseline.

An LTP-associated increase of the presynaptic release probability (PR) may facilitate cross-talk independent of astrocyte morphology changes. According to the binomial model of release, an increase of PR would decrease the variability of postsynaptic responses (coefficient of variation [CV]). Experiments using LTP induction in a single pathway showed that the CVs for the baseline AMPAR and NMDAR-mediated responses were not different between pathways and within a pathway (1/CV^2^, four paired Student t tests, p > 0.18). In addition, the rate of blockade of NMDAR-mediated response by MK801 is an indicator PR and was not affected by LTP-induction (Figure S8D).

Recordings were carried out using a Multiclamp 700B (Molecular Devices). Signals were filtered at 3-10 kHz, digitized and sampled through an AD converter (National Instruments or Molecular Devices) at 10-20 kHz, and stored for offline analysis using pClamp10 software (Molecular Devices). Receptor blockers were purchased from Tocris and Abcam Biochemicals.

#### Monte Carlo simulations

The modeling approach was described and validated against experimental data previously (Savtchenko et al., 2013; Zheng et al., 2008, 2015). In brief, the presynaptic part (Schaffer collateral en-passant boutons) and the postsynaptic part (dendritic spine heads of CA1 pyramidal cells) were represented by the two truncated hemispheres separated by a 300 nm wide 20 nm high apposition zone including a 200 nm wide synaptic cleft (Figure S7), to reflect the typical three-dimensional ultrastructure reported for these synapses (Harris et al., 1992; Lehre and Rusakov, 2002; Shepherd and Harris, 1998; Ventura and Harris, 1999). The synapse was surrounded by 20-30 nm wide extracellular gaps giving an extracellular space fraction α ∼0.15. 3000 molecules of glutamate (Savtchenko et al., 2013) were released at the cleft center and allowed to diffuse freely. The diffusion coefficient for glutamate (excluding space tortuosity due to cellular obstacles) was set at 0.4 μm^2^/ms (Zheng et al., 2008). The statistics on activation of extrasynaptic NMDARs were collected using a cluster of receptors placed at 200-250 nm from the synaptic centroid, which was approximately equidistant to the two nearest-neighboring synapses in area CA1 (Rusakov and Kullmann, 1998). To test four different scenarios pertinent to the astroglial environment of synapses, we distributed glial glutamate transporters (EAAT1-2 type) using four different patterns that occupy four sectors of the extrasynaptic environment (Figure S7). In the control case (baseline conditions) their extracellular density was ∼0.2 mM, to match a membrane surface density of 5-10⋅10^3^ μm^–2^ (Lehre and Danbolt, 1998) reported earlier. Cases (*i-iii*) thus mimicked possible astroglial re-arrangements following LTP induction. In case (*i*), the transporter density doubled while the astrocyte membrane area occupied by them was reduced two-fold (thus the total transporter number was unchanged); case (*ii*) was similar to (*i*) but with the transporter density unchanged (total number was reduced two-fold); and in the case (*iii*) the transporter-occupied area was rearranged toward one side of the nearby NMDAR cluster. During extensive control simulations we found no interaction between any of the four sectors in terms of transporter or NMDAR activation by released glutamate. In our tests, therefore, we could compare the four scenarios using the same simulations run (repeated 32 times for a statistical assessment of the stochastic receptor and transporter actions). Our simulations have suggested that, somewhat paradoxically, one factor that could prolong the presence of glutamate near NMDARs and therefore boosting receptor activation could be its stochastic unbinding from local transporters, as suggested earlier (Rusakov, 2001). Simulations were carried out using a dedicated 14-node PC cluster running under Linux (Zheng et al., 2015).

### Quantification and Statistical Analysis

The present study contained no longitudinal or multifactorial experimental designs. In electrophysiological or imaging experiments the main source of biological variance was either individual cells or individual preparations (the latter in case of field measurements in acute slices), as indicated. In accord with established practice, in the *ex vivo* tests we routinely used one cell per slice per animal, which thus constituted equivalent statistical units in the context of sampling, unless indicated otherwise. With the exception of fixed samples for EM and dSTORM studies where comparisons were made between specimens, animals or preparations provided their internal *in situ* controls. Statistical hypotheses pertinent to mean comparisons were tested using a standard two-tailed t test, unless the sample showed a significant deviation from Normality, in which case non-parametric tests were used as indicated. The null-hypothesis rejection-level was set at α = 0.05, and the statistical power was monitored to ensure that that the sample size and the population variance were adequate to detect a mean difference (in two-sample comparisons) of 10%–15% or less. Sample sizes, including the number of cells, specimens (slices), and/or animals are indicated throughout the text and figures. Group data are routinely reported as mean ± SEM, unless indicated otherwise (e.g., 95% confidence intervals, 95CI). Statistical tests were carried out using OriginPro (OriginLab).

## Supplemental Information

Document S1. Figures S1–S8

Document S2. Article plus Supplemental Information
