# Supplementary material for: LTP Induction Boosts Glutamate Spillover by Driving Withdrawal of Perisynaptic Astroglia
Source: Neuron. 2020 Dec 9;108(5):919–936.e11. doi: 10.1016/j.neuron.2020.08.030 (PMC7736499; doi:10.1016/j.neuron.2020.08.030)
Supplement: Document S1. Figures S1–S8 [file mmc1.pdf]

Neuron, Volume *108*

Supplemental Information

LTP Induction Boosts Glutamate Spillover

by Driving Withdrawal of Perisynaptic Astroglia

Christian Henneberger, Lucie Bard, Aude Panatier, James P. Reynolds, Olga Kopach, Nikolay I. Medvedev, Daniel Minge, Michel K. Herde, Stefanie Anders, Igor Kraev, Janosch P. Heller, Sylvain Rama, Kaiyu Zheng, Thomas P. Jensen, Inmaculada Sanchez-Romero, Colin J. Jackson, Harald Janovjak, Ole Petter Ottersen, Erlend Arnulf Nagelhus, Stephane H.R. Oliet, Michael G. Stewart, U. Valentin Nägerl, and Dmitri A. Rusakov

SUPPLEMENTARY FIGURES

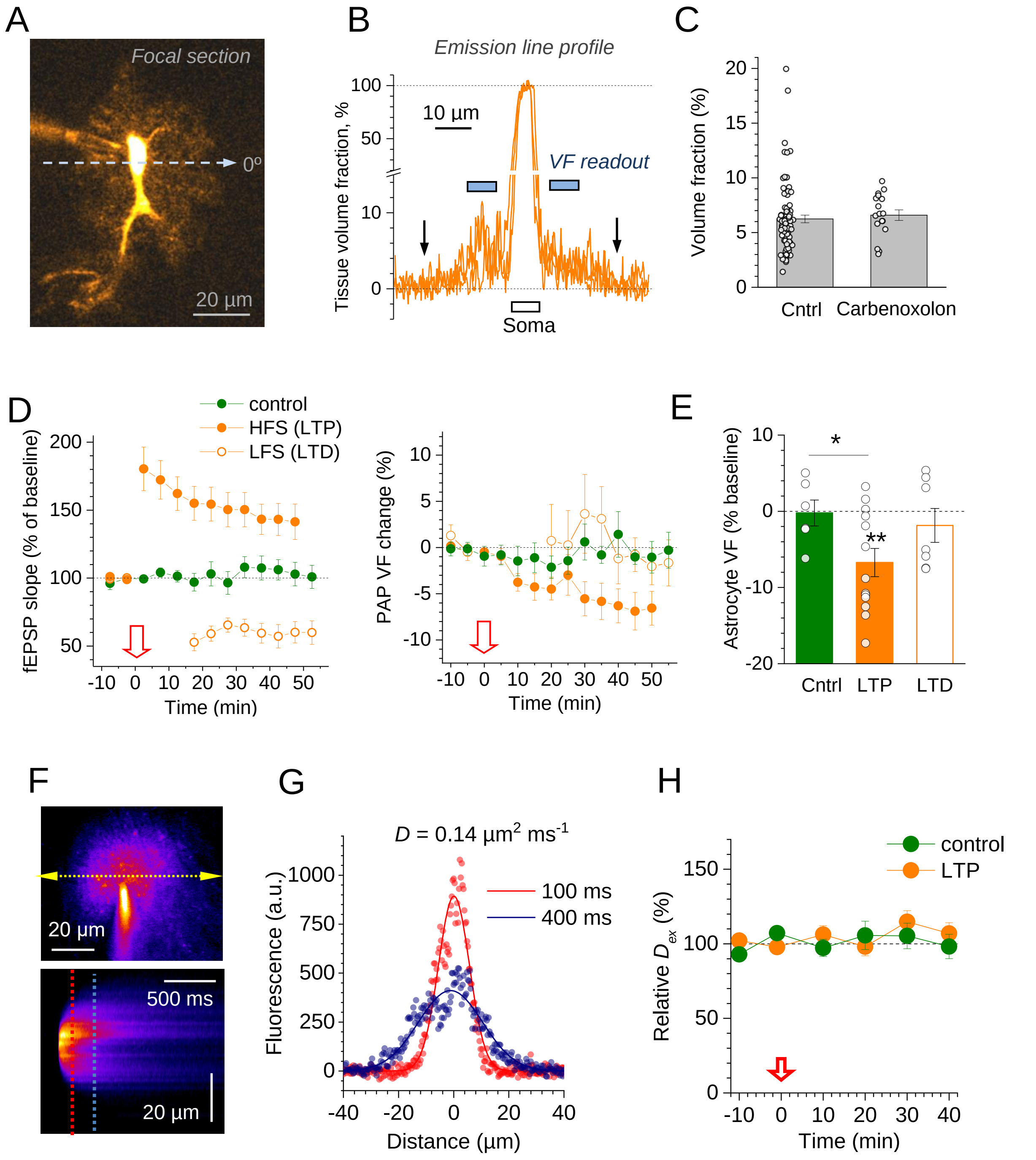

Figure S1 (related to Figure 1). PAP volume fraction and extracellular diffusivity monitored using two-photon excitation imaging.

(A) CA1 astrocyte, imaged in a single 2PE section (λ_x^{2P} = 800 nm; Alexa Fluor 594 hydrazide; 50 µM gap-junction blocker carbenoxolone added for illustration purposes); dashed line, sampling of the emission intensity profile (crossing the soma; angle 0º; false colour scale).

(B) Example of three astrocyte fluorescence profiles (measured using the original grey-level image, background-corrected): with the sampling line at 0º (shown in a), at 45º, and 135º. Data normalised to the highest intensity within the soma; arrows, detectable edges of the astrocyte profile; blue segments, typical ROI position for averaging VF readout during LTP induction (as shown in Figure 1B): ROI was selected to avoid thick primary processes near the soma or the uneven edges of the astroglial arbour.

(C) Average VF values for astrocyte arbours (soma excluded) in control conditions (mean ± SEM: 6.2 ± 0.35%, *n* = 83) were similar to those in the presence of the gap junction blocker carbenoxolone (6.6 ± 0.48%, *n* = 17, p = 0.56, unpaired *t*-test).

(D) Time course of the fEPSP slope (left) and PAP VF (right) relative to baseline (%, mean ± SEM); VF measured using astroglial EGFP expressed under a human GFAP promoter (hGFAP-EGFP); in control conditions (green dots, n = 6), during HFS LTP induction (solid orange circles; red arrow, onset; n = 13), and during the induction of LTD (low-frequency induction protocol, 1800 stimuli at 2 Hz; open orange); red arrow, onset of LTP/LTD induction.

(E) Summary of PAP VF changes (%, mean ± SEM) in experiments shown in D, 40-50 min after plasticity induction onset: LTP (- 7 ± 2%, n = 13, ** p < 0.004, *t*-test; p = 0.0081, Wilcolxon Signed Rank Test, W = 9, Z = -2.516), control (+ 0.23 ± 1.7 %, n = 6, difference with LTP at *p = 0.021), LTD (-1.9 ± 2.2 %, n = 7, p = 0.43). Note that the dynamics of PAP VF readout in EGFP-expressing cells, unlike Alexa-loaded cells, is likely to underestimate and fall behind real VF changes because diffusion equilibration of EGFP is much slower slow than that of Alexa (MW are 33 kDa and 760 Da, respectively).

(F) Measuring local extracellular diffusivity. *Top:* Snapshot of a pressurised pipette filled with Alexa Fluor 594 and placed in CA1 *S. radiatum* near the recorded astroglia; arrows, linescan position near the pipette tip. *Bottom*: Linescan recording of a brief (2-6 ms) pulse ejection of the dye; dashed lines, sampling the extracellular fluorescence intensity profiles (readout of dye concentration) at 100 ms and 400 ms post-pulse (Zheng et al., 2008).

(G) Fluorescence intensity profiles (dye concentration readout, shown in F) approximated with a Gaussian, as indicated. Effective extracellular diffusion coefficient *D* is obtained from the time evolution of such profiles, as described earlier (Zheng et al., 2008).

(H) Time course of extracellular diffusivity in experiments with and without LTP induction (arrow), as indicated; average extracellular diffusivity 40 min after LTP induction (mean ± SEM): 107.0 ± 7.1% of baseline (*n* = 8); 40 min time point in control (no-LTP) experiments was 98.2 ± 8.2% of baseline (*n* = 6).

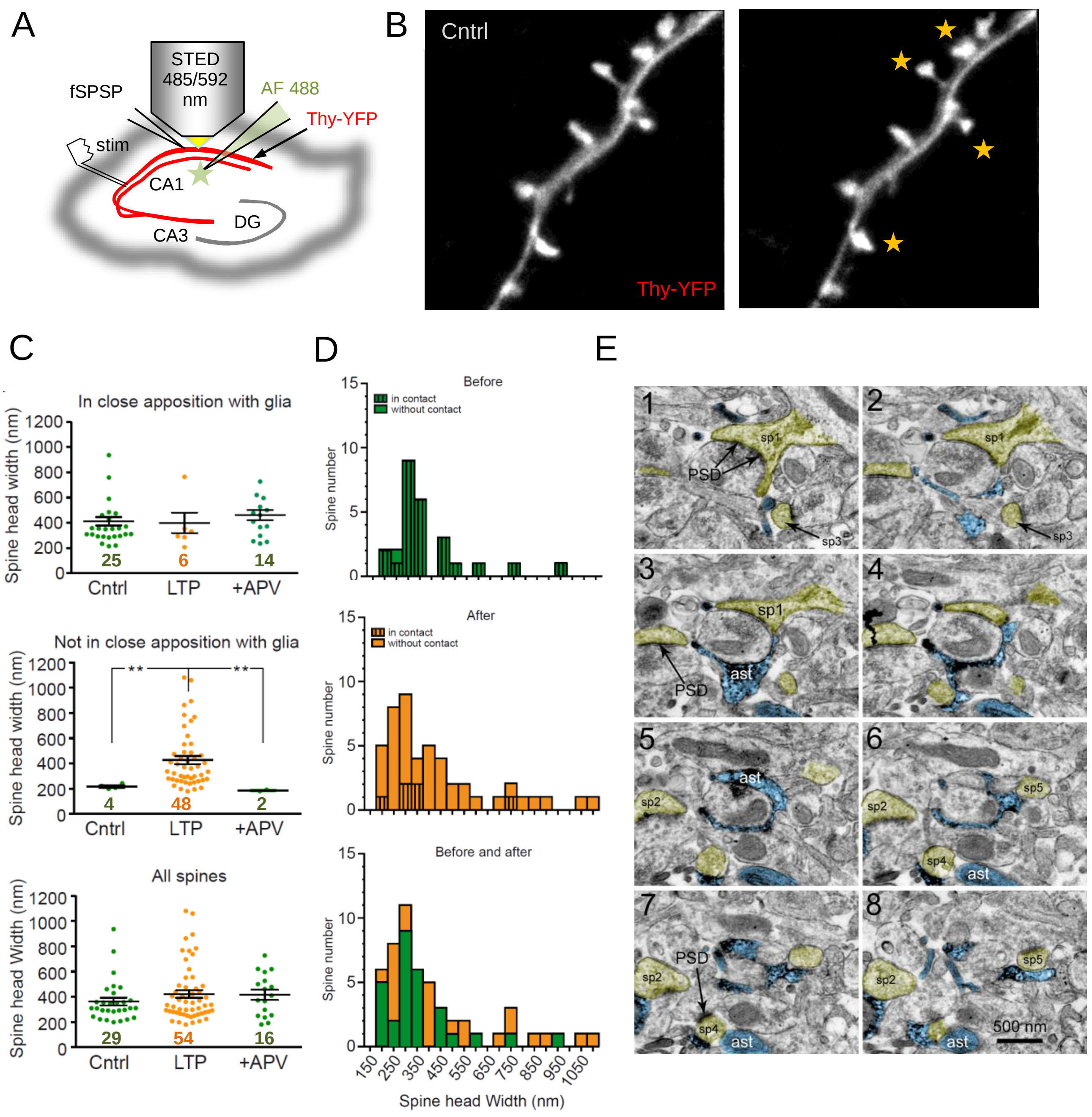

Figure S2 (related to Figure 2). STED imaging and 3D EM of LTP-associated changes in perisynaptic astroglial environment.

(A) Experiment diagram: CA3-CA1 LTP is induced in organotypic hippocampal slices, with Thy-YFP-labelled CA1 pyramidal cells (red channel) and whole-cell recorded astroglia (Alexa Fluor 488, green channel) for STED imaging.

(B) Characteristic STED images of CA1 pyramidal cell dendritic spines (optimised Thy1-YFP channel), before and ~25 min after LTP induction, as indicated (STAR Methods). Stars indicate spine heads showing visible shape alterations after LTP induction.

(C) Effects of LTP induction on the occurrence of close astroglia-spine apposition and on the dendritic spine head sizes, monitored with STED microscopy. Digits, numbers of identified spine heads under pseudo-random inspection of the area of interest; bars, mean ± SEM; LTP, data ~25 min after LTP induction; +APV, same protocol but in the presence of 50 µM APV; ** $p < 0.01$ (Kruskall-Wallis ANOVA, sample sizes *n* shown).

(D) Frequency histograms of spine head sizes before and 20-25 min after LTP induction (data set as in C), also with the separation between spines which are closely approached (striped columns) and not approached (plain columns) by PAPs, as shown.

(E) 3D electron microscopy: Example of serial sections containing profiles of the astroglia recorded and labelled in an acute slice; eight (1-8) 60 nm thick adjacent serial sections are shown, depicting dendritic spines (yellow shade; sp1-sp5) and astroglia (blue, ast; filled with electron dense precipitate from DAB conversion of the recorded astrocyte filled with biocytine); scale bar, 500 nm.

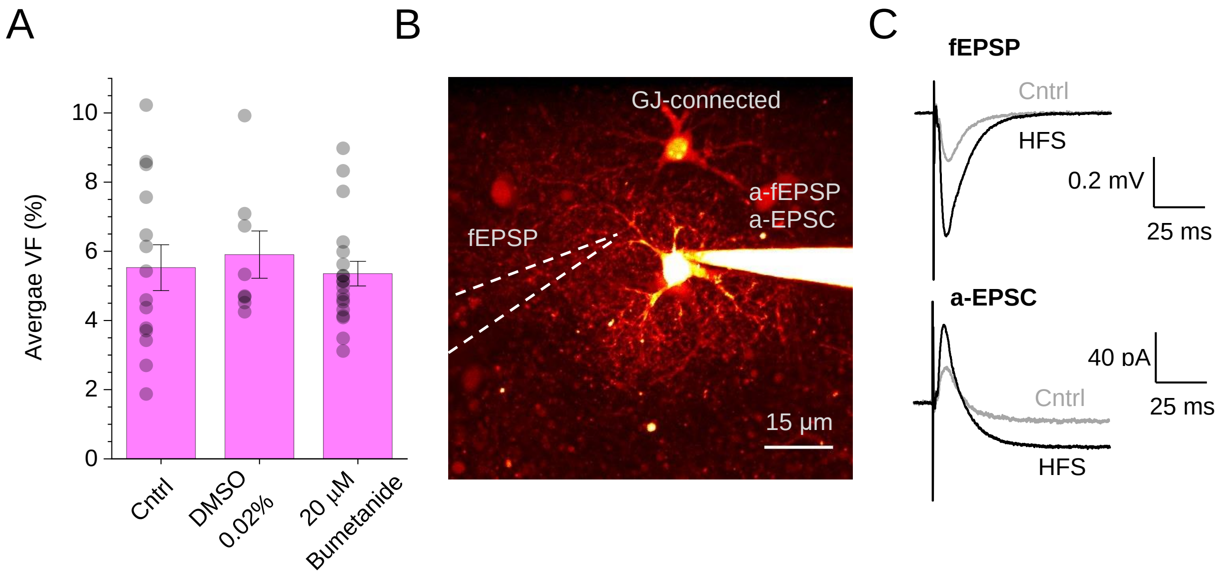

Figure S3 (related to Figure 3). Control tests for astroglial VF in bumetanide and LTP induction with S3 peptide.

(A) Astroglial PAP VF (%, mean ± SEM) measured in control conditions (Cntrl, 5.52 ± 0.66; n = 14), with the vehicle 0.02% DMSO (5.90 ± 0.68; n = 8), and with 20 µM bumetanide inside the cell (5.35 ± 0.36; n = 19).

(B) Experimental arrangement in LTP experiments (including S3 peptide dialysis), in acute slices, area CA1 *S. radiatum*; Alexa Fluor 594 channel, λ_x^{2P} = 800 nm. Locations of the whole-cell patch pipette (recording astroglial a-fEPSPs or a-EPSCs), extracellular electrode (recording fEPSPs), and gap junction connected astroglia (GJ-connected) are indicated.

(C) Characteristic recordings before and ~20 min after high-frequency stimulation (HFS) induced LTP using an extracellular electrode (fEPSP) and an astrocyte pipette (a-EPSC) (Henneberger and Rusakov, 2012), in the same peptide S3 experiment.

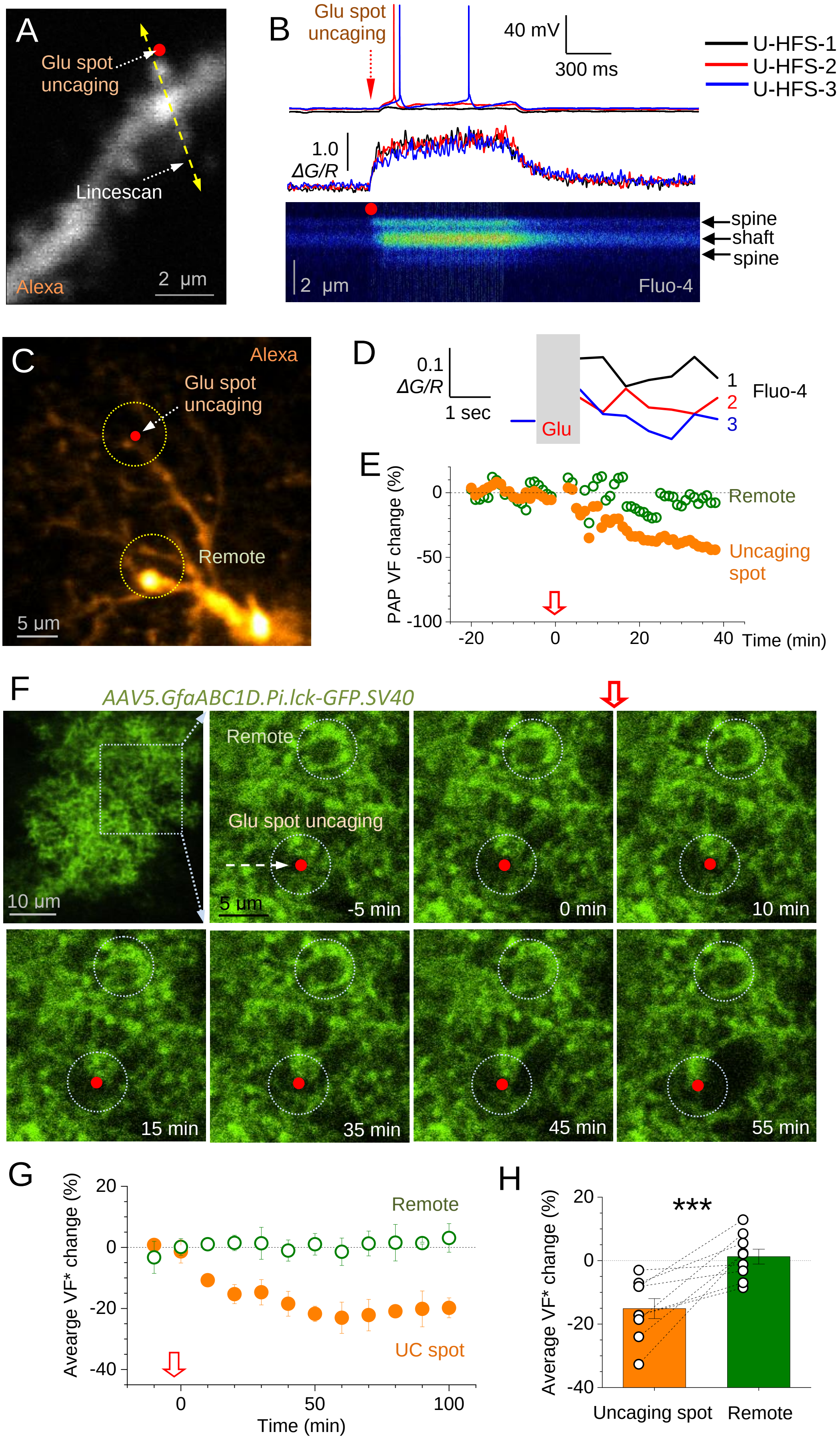
A
Glu spot uncaging
Lincescan
Alexa
2 μm
B
Glu spot uncaging
40 mV
300 ms
U-HFS-1
U-HFS-2
U-HFS-3
1.0
ΔG/R
spine
shaft
spine
2 μm
Fluo-4
C
Alexa
Glu spot uncaging
Remote
5 μm
D
0.1
ΔG/R
1 sec
Glu
1
2
3
Fluo-4
E
PAP VF change (%)
0
-50
-100
-20
0
20
40
Time (min)
Remote
Uncaging spot
F
AAV5.GfaABC1D.Pi.lck-GFP.SV40
10 μm
Remote
Glu spot uncaging
5 μm
-5 min
0 min
10 min
15 min
35 min
45 min
55 min
G
Average VF* change (%)
20
0
-20
-40
Remote
UC spot
0
50
100
Time (min)
H
Average VF* change (%)
20
0
-20
-40
***
Uncaging spot
Remote

Figure S4 (related to Figure 4). LTP induction by glutamate spot-uncaging induces postsynaptic depolarisation and Ca^{2+} entry while triggering local PAP VF reduction.

(A) Dendritic fragment, CA1 pyramidal cell (Alexa channel); red dot, MNI-glutamate uncaging spot; yellow arrow, linescan position; whole-cell current-clamp mode (V_m = -60...65 mV in baseline conditions); uncaging at λ_u^{2P} = 720 nm; Alexa imaging at λ_x^{2P} = 840 nm.

(B) Somatic voltage recording (upper traces) and postsynaptic Ca^{2+}-sensitive linescan recording (bottom traces, Δ*G/R;* linescan, Fluo-4 channel) during LTP induction (100 x 1 ms laser pulses at 100Hz, three series 60 s apart), three traces superimposed (colour-coded; U-HFS-1/2/3); intermittent spikes can be seen in some traces.

(C) Astrocyte fragment (averaged 9-section z-stack) depicting the uncaging spot (red dot) and two ROIs for PAP VF measurement; Alexa Fluor 594 channel.

(D) Time course of Ca^{2+}-sensitive fluorescence (averaged within ROI1), before and immediately after 2P spot-uncaging (grey segment), for series of pulses 1, 2, and 3, as shown in B.

(E) Relative change in PAP VF (%) in ROI1 and ROI2 shown in C during LTP induction (red arrow, onset).

(F) One-cell example of PAP changes during LTP induction with glutamate spot-uncaging; the astrocyte-membrane bound GFP (transduction with AAV5.GfaABC1D.Pi.lck-GFP.SV40) provides readout of PAP (membrane) presence within ROI (PAP VF*, integrated brightness within ROI). Top left panel: astrocyte view; dotted rectangle, monitored area (10 µm deep z-stack average). Other panels: time-lapse images of the monitored area (single 2PE focal section, time stamps shown), during LTP induction (red arrow, onset); circles, ROIs for measuring PAP VF* near the uncaging spot and within a control / remote area (Remote), as indicated; acute hippocampal slice, *S. radiatum*.

(G) Time course of PAP VF* change relative to baseline (%, mean ± SEM, n = 8 slices) in the experiments illustrated in (F).

(H) Summary of experiments shown in (F-G), for the PAP VF* changes over 25-35 min after LTP induction; bar graphs, mean ± SEM (n = 8); connected dots, individual experiments; ***, $p < 0.001$ (paired *t*-test).

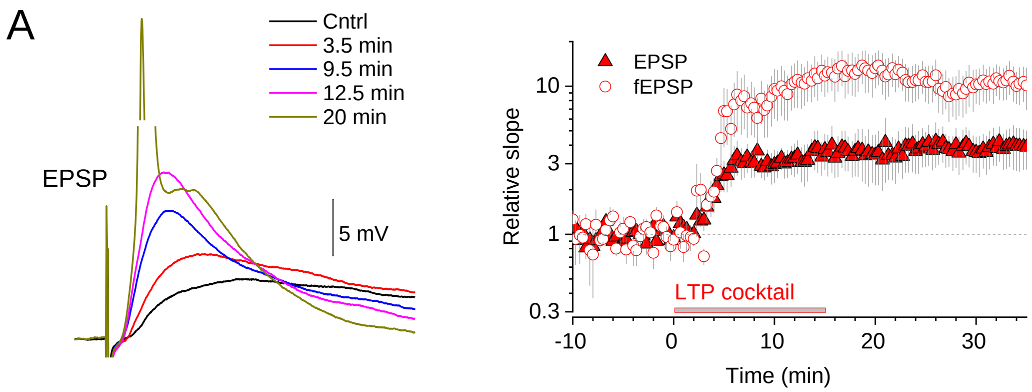

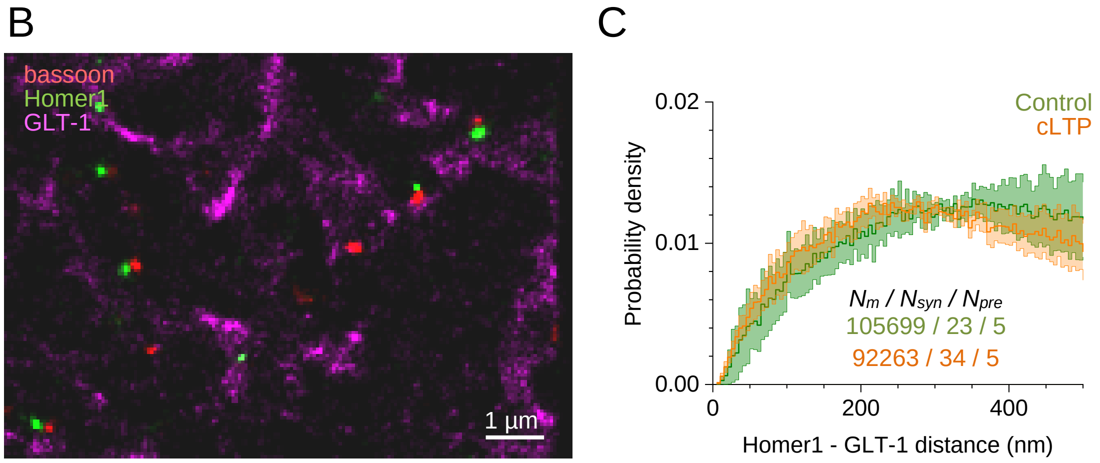

D

DNA:

start
His-tag T7-tag Xpress-tag Biotin-tag flexible linker(GSSG) after biotin tag eCFP GltI Venus
stop

Protein:

```
MRGSHHHHHHGMASMTGGQQMGRDLYDDDDKDPKLKVTVNGTAYDVDVDVDKSHENPMGTILFGGGTGGAPAPAAGGAGAGKAGEGEIPAPLAGTVSKILVKEGD
TVKAGQTVLVLEAMKMETEINAPTDGKVEKVLVKERDAVQGGQGLIKIGDLELIEGSSGSDPGRMVSKGEELFTGVVPILVELDGDVNGHKFSVSGEGEGDATYG
KLTLKFICTTGKLPVPWPTLVTTLTWGVQCFSRYPDHMKQHDFFKSAMPEGYVQERTIFFKDDGNYKTRAEVKFEGDTLVNRIELKGIDFKEDGNILGHKLEYNY
ISHNVYITADKQKNGIKANFKIRHNIEDGSVQLADHYQQNTPIGDGPVLLPDNHYLSTQSALSKDPNEKRDHMVLLEFVTAAGITLGMDELYKGGTGGAAGSTLD
KIAKNGVIVVGHRESSVPFSYYDNQQKVVGYSQDYSNAIVEAVKKKLNKPDLQVKLIPITSQNRIPLLQNGTFDFECGSTTNNVERQKQAAFSDTIFVVGTRLLT
KKGGDIKDFANLKDKAVVVTSGTTSEVLLNKLNEEQKMNMRIISAKDHGDSFRTLESGRAVAFMMDDALLAGERAKAKKPDNWEIVGKPQSQEAYGCMLRKDDPQ
FKKLMDDTIAQVQTSGEAEKWFDKWFKNPIPPKNLNMNFELSDEMKALFKEPNDKALNGAGTGGMVSKGEELFTGVVPILVELDGDVNGHKFSVSGEGEGDATYG
KLTLKLICTTGKLPVPWPTLVTTLGYGLQCFARYPDHMKQHDFFKSAMPEGYVQERTIFFKDDGNYKTRAEVKFEGDTLVNRIELKGIDFKEDGNILGHKLEYNY
NSHNVYITADKQKNGIKANFKIRHNIEDGGVQLADHYQQNTPIGDGPVLLPDNHYLSYQSALSKDPNEKRDHMVLLEFVTAAGITLGMDELYK-
```

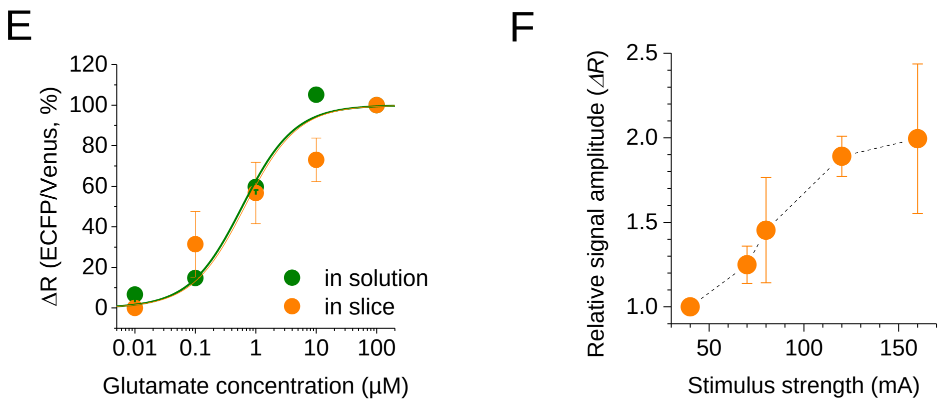

Figure S5 (related to Figure 5). LTP-associated perisynaptic withdrawal of glutamate uptake system documented with super-resolution dSTORM and extracellular glutamate sensor bFLIPE600n.

(A) Induction of chemical long-term potentiation (cLTP) in acute hippocampal slices. Traces, characteristic EPSPs (whole-cell current clamp) recorded in CA1 pyramidal cells in response to electrical stimulation of Schaffer collaterals, in control conditions (Cntrl), and at different time points after the onset of 'LTP cocktail' application (50 µM forskolin and 100 nM rolipram) (Otmakhov et al., 2004), as indicated. Graph, average fEPSP slope and whole-cell EPSPs relative to baseline (mean ± SEM, n = 8 slices) during cLTP induction.

(B) 3D three-color dSTORM: a snapshot depicting molecular patterns of presynaptic bassoon (CF-568, red), postsynaptic Homer 1 (Atto-488, green), and glutamate transporter GLT-1 (Alexa-647, magenta), in the *S. radiatum* (acute slices, 1-month-old rats, control conditions); images show 2D projections of 3D dSTORM molecular maps (~4 µm deep stacks) in 30-µm acute slices; label brightness reflects molecular density, high-resolution raw data include single-molecule 3D co-ordinates.

(C) Average distribution (probability density, mean ± SEM) of the nearest-neighbour distances (<500 nm) between GLT-1 and postsynaptic Homer1 molecules, in control and potentiated tissue (~30 min after 'chemical' LTP induction; STAR Methods), as indicated; summary for N_m inter-molecular distances at N_{syn} synapses from N_{pre} individual slices; SEM reflects variance among N_{pre} = 5 slices.

(D) Glutamate sensor bFLIPE600n: DNA sequence groups, and the protein sequence. Functionally relevant parts are colour-coded, as indicated.

(E) Measuring glutamate sensitivity of bFLIPE600n immobilised in acute hippocampal slices. Glutamate levels were estimated by calculating the fluorescence intensity ratio R = ECFP/Venus and its changes (ΔR). Titration of bFLIPE600n was performed in free solution and acute slices (ΔR versus nominally zero glutamate; free solution: K_d = 596 nM, n = 3; acute slices K_d = 659 nM, n = 5). *In situ* measurements were performed in the presence of 10 µM NBQX, 50 µM D-APV, 1 µM TFB-TBOA, 1 µM TTX, 50 µM LY341495. Calibration curves, Hill equation approximation (Hill coefficient set to one).

(F) The input-output evaluation bFLIPE600n sensitivity: glutamate transient amplitude versus stimulation intensity (mean ± SEM, n = 4) in CA1 *S. radiatum*; ΔR signal is normalized to the baseline response at 40 µA stimulus.

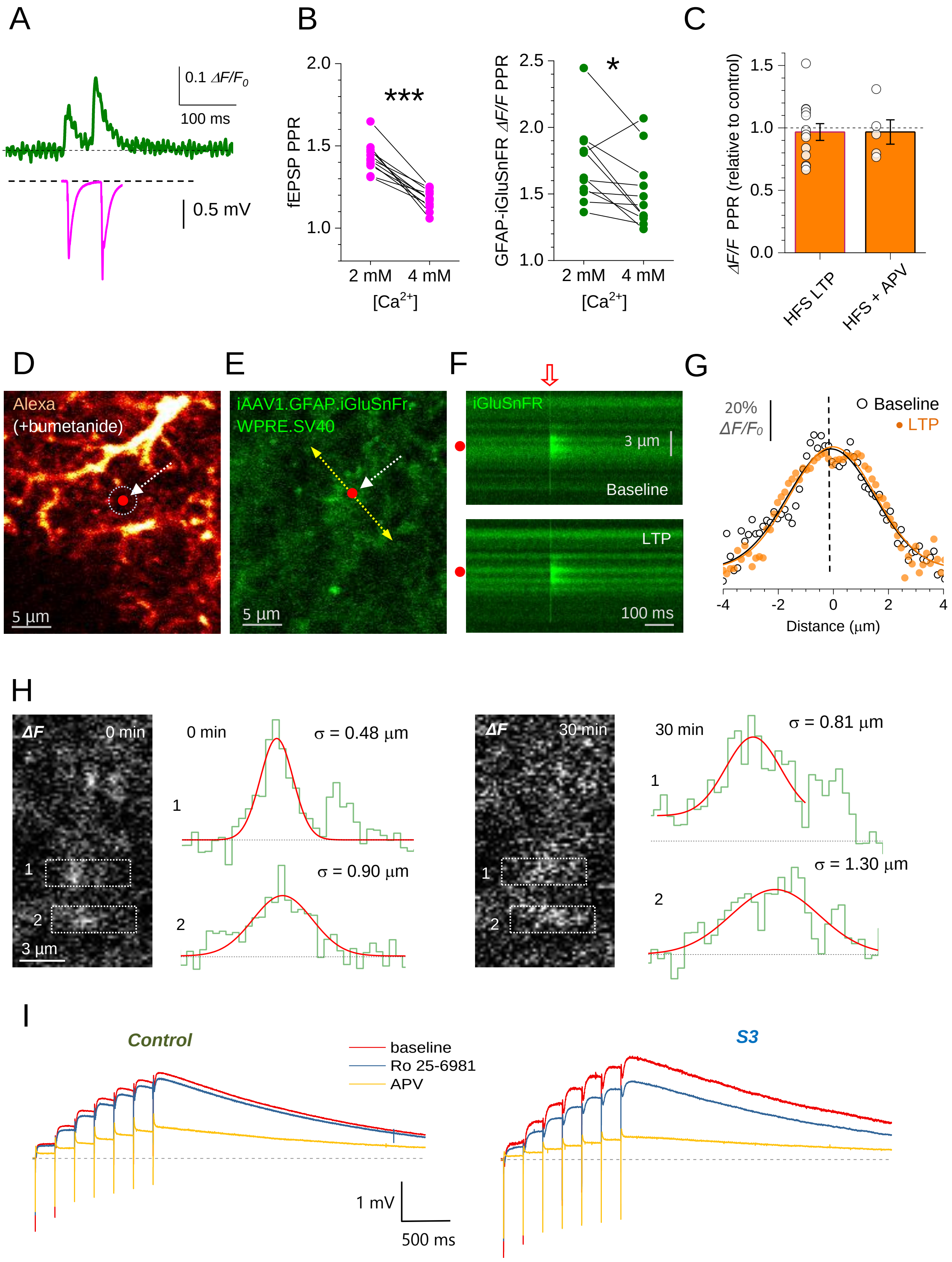

Figure S6 (related to Figure 6). Monitoring extrasynaptic glutamate escape with the optical glutamate sensor iGluSnFR and through activation of extrasynaptic NMDA receptors.

(A) iGluSnFR fluorescence intensity traces ($\Delta F/F_0$) during paired-pulse afferent stimuli (50 ms apart; top trace, average of 100 trials) faithfully reflect fEPSPs recorded in parallel (bottom trace).

(B) Paired-pulse experiments (as in A) showing that increasing extracellular [Ca^{2+}] reduces paired-pulse ratio (PPR) for both fEPSPs (left; $p < 0.001$) and optical iGluSnFR responses (right; $p = 0.019$; $n = 11$; paired *t*-tests).

(C) HFS-induced LTP (n = 13), or HFS in the presence of 50 µM APV (n = 5), has no detectable effect on the paired-pulse ratio (PPR) of iGluSnFR $\Delta F/F$ responses.

(D) Fragment of the astrocyte (whole-cell loaded with 100 µM Alexa Fluor 594 and 20 µM bumetanide; λ_x^{2P} = 910 nm; single optical section) illustrating the LTP induction protocol with spot-uncaging of glutamate (red dot; λ_u^{2P} = 720 nm); dotted circle, ROI for monitoring local PAP VF during LTP induction. See Figure 6D for statistical summary.

(E) Fragment in d shown in the iGluSnFR channel (λ_x^{2P} = 910 nm); arrow, linescan position; other notations as in D.

(F) Examples of linescan traces (positioned as in E) recorded before and ~20 min after the LTP induction spot-uncaging protocol (baseline and LTP, respectively).

(G) Spatial glutamate-sensitive iGluSnFR fluorescence profiles (dots, individual pixel values) evoked by a 1 ms glutamate uncaging pulse, before (Baseline) and 20-25 min after LTP induction, as indicated, in the experiment illustrated in D-F; zero abscissa, the uncaging spot position (red dot in D-E); black and orange solid lines, best-fit Gaussian approximation. See Figure 6D for statistical summary.

(H) Example illustrating evaluation of the axonal glutamate signal spread in the experiment shown in Figure 6E-F, just before (0 min) and 30 min after LTP induction, as indicated. Image panels, grey-level images of the $\Delta F = F - F_0$ signal (AAV9.hSynap. iGluSnFr.WPRE.SV40 fluorescence landscape obtained by subtracting the baseline landscape from that during five afferent stimuli at 20Hz); dotted rectangles, sampling (horizontal) segments to acquire brightness profiles associated with tentative axonal boutons 1 and 2, as indicated; plots, the corresponding brightness profiles (staircase line, green) with the best-fit Gaussian (red); σ, Gaussian standard deviation (FWHM = 2.35σ). Note that in the Bouton 1 Gaussian fitting ignores the neighbouring bouton (a second fluorescence peak to the right).

(I) Examples of CA1 astrocyte-recorded fEPSPs (a-fEPSP, whole-cell current clamp, raw traces related to Figure 6I) evoked by seven Schaffer collateral stimuli (5 Hz), in baseline conditions and after blocking GluN2B-containing NMDARs with 1 µM Ro 25-6981, followed by application of 50 µM APV, in control tests and with 1 µM peptide S3 inside the patched astrocyte, as indicated.

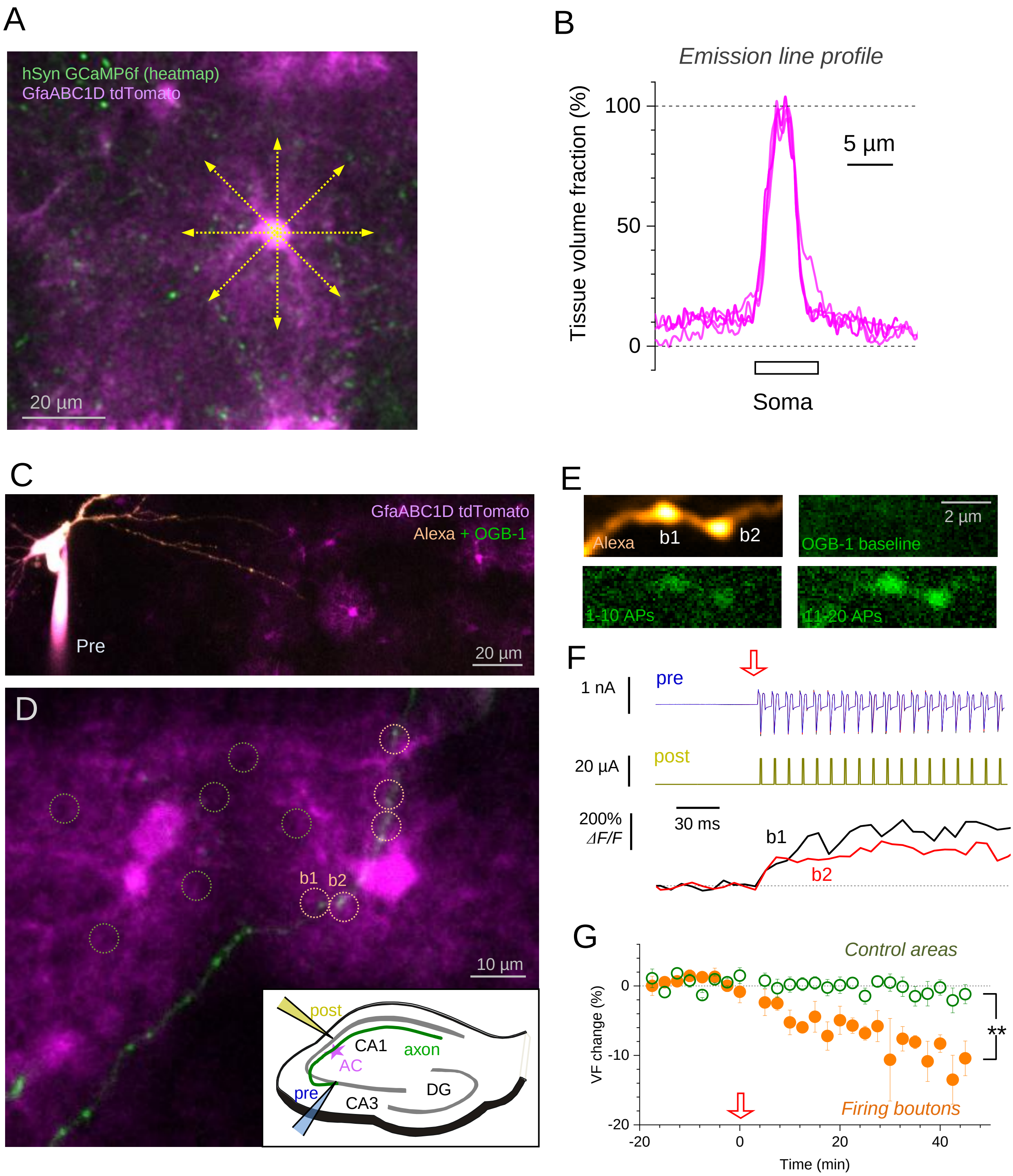

Figure S7 (related to Figure 7). Testing VF readout in tdTomato labelled astroglia: LTP pairing protocol for a single CA3 pyramidal cell axon leads to astroglial withdrawal near active axonal boutons.

(A) Barrel cortex view (S1BF) through the cranial window (live image, λ_x^{2P} = 1040 and 910 nm, single focal section, fragment of image in Figure 7D); dashed arrows, example

of brightness-profile sampling lines at 0º, 45º, 90º, and 135º centred at a selected astrocyte soma.

(B) Astroglial VF profile along the sampling lines shown in (A) (relative to somatic signal set as 100%); the profile is similar to that in astroglia loaded whole-cell with Alexa Fluor (Figure S1B).

(C) CA3 pyramidal cell held in whole-cell (dialysed with 50 µM Alexa 594 and 200 µM OGB-1), with the axon proximal part seen traced into the field of astrocytes labelled with tdTomato (magenta); a 67 µm deep z-stack projection image (two-laser 2PE at λ_x^{2P} = 800 nm and λ_x^{2P} = 910 nm).

(D) A distal fragment of the same axon (green) which crosses local CA1 astroglia (magenta); orange dotted circles, ROIs for PAP VF monitoring near five firing presynaptic boutons (see below for Ca^{2+} recordings in boutons b1 and b2); green dotted circles, examples of control astroglial areas devoid of the presynaptic axon; a 67 µm deep z-stack projection image for two-laser 2PE as in C.

Inset, experiment diagram of LTP induction protocol by pairing, depicting presynaptic cell held in whole-cell (as in C), a postsynaptic stimulating electrode placed in *stratum pyramidale* (yellow; to depolarize and/or fire postsynaptic cells), a single traced axon (green) crossing an astrocyte (AC, magenta).

(E) Examples of two presynaptic axonal boutons (b1 and b2 in D), shown in the Alexa channel (red) and in the Ca^{2+} sensitive OGB-1 channel (green) in baseline conditions and following the initial burst of presynaptic action potentials Nos 1-10, and 11-20 (at 100 Hz), as indicated.

(F) Examples of presynaptic whole-cell electrode recordings (blue, individual evoked spikes shown), postsynaptic extracellular electrode pulse control (dark yellow), and OGB-1 fluorescence in boutons b1 and b2 (shown in D-E), in the initial phase of the LTP induction protocol (3 x 1 s trains @ 100 Hz; red arrow, onset).

(G) Time course of PAP VF changes (%, mean ± SEM) near firing axonal boutons (orange dots, the corresponding ROIs shown by orange circles in D; n = 5) and in control areas (ROI examples shown by green circles in D; n = 10) during LTP induction (red arrow, onset); **, $p < 0.01$ (two-sample *t*-test for 25-45 min interval post-induction); the data were routinely adjusted to account for the (small) experiment-wide tdTomato photobleaching, which was fitted by $P(t) = 0.9723 + 0.00867\, e^{-t/\tau}$, with τ = 10.9 min.

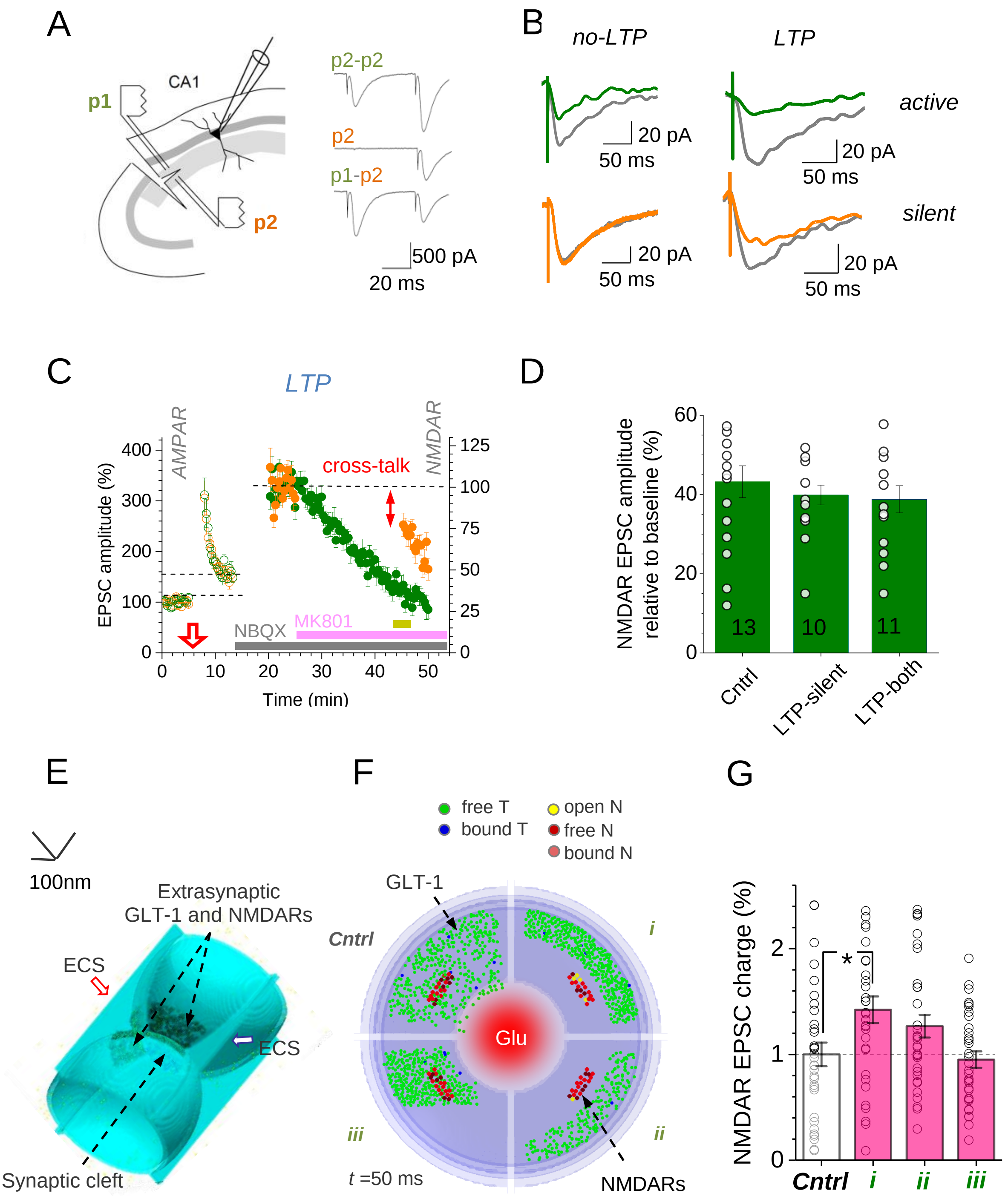

Figure S8 (related to Figure 8). Electrophysiological probing of NMDAR-mediated inter-synaptic cross-talk and a biophysical plausibility test.

(A) Experiment diagram (left), a previously established test (Scimemi et al., 2004) for presynaptic independence of two Schaffer collateral pathways (p1 and p2) converging onto a CA1 pyramidal cell. Traces, EPSCs show paired-pulse facilitation during p2-p2 stimulation but not across the pathways (p1-p2, modified from (Scimemi et al., 2004)). In the recorded sample (*n* = 54 slices), paired-pulse facilitation with a 50 ms interval

was 75.4 ± 6.1 % (mean ± SEM; $p < 0.001$) in the same pathway and only 16.5 ± 2.9% across (difference at $p < 0.001$).

(B) Characteristic one-cell examples (summaries shown in Figure 8A-B) of NMDAR EPSCs recorded in both pathways, in baseline conditions (grey) and after resuming stimulation of the silent (green) pathway; in baseline conditions (*no-LTP*) and with LTP induced in the active (orange) pathway during AMPA EPSC recording (*LTP*).

(C) Summary of two-pathway experiments with LTP induced simultaneously in both (rather than one) pathways. Notations are as in Figure 8A; yellow segment, period over which the degree of NMDAR EPSC suppression by MK801, prior to the resumption of silent pathway stimulation, was measured.

(D) The reduction of the NMDAR EPSC amplitude (mean ± SEM, sample size n shown) following application of MK801, prior to the resumption of test pathway stimulation (averaging time interval depicted by yellow segment in C), in experiments with no LTP, with LTP induced in the silent pathway (LTP-silent), and with LTP induced in both pathways (LTP-both), as indicated. See Figure 8A for the entire time course data. No difference in the MK801-induced decay among the three conditions indicate no difference in release probabilities in the bulk of stimulated synapses, with or without LTP induction.

(E) A 3D Monte-Carlo model depicting the microenvironment of the CA3-CA1 synapses. Truncated hemispheres represent surfaces of the presynaptic bouton and the postsynaptic dendritic spine separated by the synaptic cleft and surrounded by extracellular space gaps (ECS). Extrasynaptic clusters of astroglial transporters (GLT-1, on the PAP surface) and NMDA receptors (on the surface of dendrites facing PAPs) are shown (STAR Methods) (Zheng et al., 2008).

(F) Front view (cross-section) of the model shown in E. For illustration purposes, four scenarios of astroglial GLT-1 relocation (green dot scatter) during LTP are shown in four different quadrants: *Cntrl* (random scatter), *i* (even withdrawal/shrinkage, same transporter numbers), *ii* (even withdrawal, same transporter density, ~50% drop in numbers), *and iii* (withdrawal to one side, same transporter numbers). Images illustrate a snapshot of NMDAR activation 50 ms following release of 3000 glutamate molecules from the centre (red shade); GLT-1 transporter (T) and NMDAR (N) states are color-coded, as indicated (Video S4).

(G) Summary of Monte Carlo experiments (n = 32 runs) shown in (E-F). Average NMDAR-mediated charge transfer relative to Control (*Cntrl*), for the three types of change: *i* (mean ± SEM; 1.42 ± 0.13, $p < 0.015$ compared to control), *ii* (1.26 ± 0.11), and *iii* (0.95 ± 0.08); dots, data points at individual runs; note high variability due to a few NMDARs being activated stochastically per release. The data indicate that scenario *i* is most likely to correspond to the larger increase in extrasynaptic NMDAR activation after LTP induction. Note that the higher transporter density in scenario *i*, if compared to

scenario *ii,* prolongs local dwell time of glutamate molecules, due to unbinding form transporters.
